# Clinical and Socioeconomic Burden of RSV Infections Among Older Adults in Primary Care: An International Prospective Cohort Study

**DOI:** 10.1111/irv.70174

**Published:** 2025-10-11

**Authors:** Sarah F. Hak, Joanne G. Wildenbeest, Sara Bracaloni, Michela Scarpaci, Tommaso Cosci, Enrica Esposito, Maria Chironna, Donatella Panatto, Giancarlo Icardi, Melissa Torrisi, Leonardo Bonaldo, Eugenio Mammolenti, Emma te Bogt, Jacqueline Vlaskamp‐Smit, Louis J. Bont, Caterina Rizzo, Roderick P. Venekamp

**Affiliations:** ^1^ Department of Paediatric Infectious Diseases and Immunology Wilhelmina Children's Hospital/University Medical Center Utrecht Utrecht Netherlands; ^2^ Department of Translational Research and New Technologies in Medicine and Surgery University of Pisa Pisa Italy; ^3^ Department of General Practice & Nursing Science, Julius Center for Health Sciences and Primary Care University Medical Center Utrecht, Utrecht University Utrecht Netherlands

**Keywords:** burden, costs, incidence, older adults, outpatient, primary care, respiratory syncytial virus, RSV

## Abstract

**Introduction:**

Respiratory syncytial virus (RSV) is increasingly recognized as an important cause of acute respiratory infections (ARI) in older adults. However, primary care data on RSV infections are scarce.

**Methods:**

We conducted a prospective cohort study over two winter seasons (2022–2023 and 2023–2024) in Italy and the Netherlands (NCT06318936). Older adults (≥ 60 years) presenting to primary care with ARI were tested for RSV and influenza. Clinical and socioeconomic burden was assessed through questionnaires on Days 1, 14, and 30. In secondary analyses, we compared between RSV‐ and influenza‐positive patients and estimated RSV‐ARI incidence in Dutch primary care.

**Results:**

Of 703 older adults tested, 93 (13.2%) were RSV‐positive and 100 (14.2%) influenza‐positive. In RSV patients (mean age: 76 years [SD: 8], 63% ≥ 1 comorbidity), mean illness duration was 17 days (SD: 10). Repeat primary care visits occurred in 38% (33/87), emergency department referral in 5% (4/88), and hospitalization in 2% (2/88) of RSV patients. The mean costs per RSV episode were €78.1 (95%CI: 74.4–81.8) and €279.7 (95%CI: 245.5–318.2) from a healthcare system and societal perspective, respectively. The annual RSV‐ARI incidence rate was 10.3 episodes per 1000 person‐years. RSV patients were significantly older, and had less often fever, muscle pain, and fatigue than influenza patients, but clinical and socioeconomic burdens were comparable.

**Conclusions:**

This prospective study is the first sufficiently large to demonstrate that the primary care burden of RSV infections among older adults is substantial and comparable with influenza. These findings are highly relevant for informing public health decisions on novel RSV vaccines.

## 1 Introduction

Respiratory syncytial virus (RSV) is increasingly recognized as an important cause of acute respiratory infections (ARI) among older adults [1]. RSV affects approximately 3%–7% of community‐dwelling older adults (≥ 60 years of age) each year, with 17%–28% of symptomatic cases seeking medical care [2, 3, 4]. As such, RSV may account for around 10% of all ARI‐related doctor visits in older adults during winter [5, 6]. It is estimated that 5.2 million older adults in high‐income countries suffer from RSV‐associated ARI annually, leading to nearly 470,000 hospital admissions [7]. Despite most RSV infections in older adults being managed in outpatient settings [8, 9], the clinical and socioeconomic burden of these infections is largely unknown.

Previous community and outpatient‐based studies have primarily focused on incidence and prevalence of RSV ARI, rather than its impact on the individual patient and society at large. Only few assessed the clinical and societal burden over time, including illness duration [2, 4], impact on daily life [8], healthcare visits [6, 8], work absence, and associated costs [10]. Notably, these studies were often limited by small number of RSV cases [2, 4, 10], primarily conducted in the United States [4, 6, 9], or relied on the influenza‐like illness (ILI) case definition [11, 12] which may underdetect RSV [13], thus underestimating its true burden. Moreover, all studies were conducted prior to the COVID‐19 pandemic, which may have altered viral transmission dynamics [14]. Hence, there is need for updated, detailed data from European primary care settings. These data are particularly relevant for evaluating cost‐effectiveness of newly approved RSV vaccines for older adults [15, 16, 17, 18].

Because influenza is a well‐recognized cause of ARI among older adults, which shares seasonal patterns and clinical features with RSV, comparative data can provide valuable context to better understand the relative impact of RSV [1].

We therefore conducted a prospective cohort study among older adults seeking primary care with ARI to assess the clinical and socioeconomic burden of RSV infections, and compared these outcomes with those caused by influenza.

## 2 Methods

### 2.1 Study Design and Population

This prospective multi‐centre cohort study (ClinicalTrials.gov identifier: NCT06318936) was conducted across two consecutive RSV seasons (2022–2023 and 2023–2024, November–March) in primary care practices in Italy and the Netherlands, respectively. Detailed information on the primary care systems in both countries is provided in the Supporting Information.

All adults aged ≥ 60 years presenting to their general practitioner (GP) with ARI symptoms during regular office hours were offered RSV and influenza testing by nasopharyngeal swab. For ARI, we adhered to the World Health Organization definition for community‐based surveillance, that is, acute onset (within last 10 days) of ≥ 1 symptom: shortness of breath, coughing, wheezing, sore throat, or coryza [19]. Exclusion criteria were previous or current participation in RSV interventional trials and inability to provide informed consent (e.g., due to cognitive impairment or insufficient proficiency of national or English language to understand study information).

### 2.2 Study Procedures

Nasopharyngeal swabs from eligible older adults were obtained by the GP and tested for RSV and influenza A/B, using multiplex real‐time polymerase chain reaction (Allplex Respiratory Full Panel Assay) in Italy and a point‐of‐care molecular test (Abbott ID NOW) in The Netherlands.

RSV‐positive older adults were asked to provide consent for follow‐up over a 30‐day period after the initial primary care consultation. Data were collected at three time points to minimize potential recall bias. On Day 1 (GP visit), GPs completed a short clinical report, including relevant medical history and presenting symptoms. On Days 14 and 30, participants completed a questionnaire, either via telephone or digitally, which included questions regarding symptoms, healthcare and medication utilization, and health‐related quality of life (HRQoL). HRQoL was assessed using the EQ‐5D‐5L tool [20, 21], which consists of a descriptive system with five dimensions (Mobility, Selfcare, Usual Activities, Pain/Discomfort, Anxiety/Depression) and a visual analogue scale [22]. In the Netherlands, influenza‐positive patients were also asked to consent for 30‐day follow‐up data collection.

### 2.3 Outcomes and Analysis

#### 2.3.1 Clinical Burden

The RSV clinical disease burden was captured using the following metrics: RSV positivity rate, patient characteristics (e.g., demographics, comorbidities), disease characteristics (symptoms, illness duration), healthcare resource utilization (primary care visits, ED referrals, hospitalization rate, medication use), and HRQoL impact.

Clinical disease burden outcomes were presented both overall, and stratified by age (60–74 years and ≥ 75 years), and country. Categorical variables were summarized as frequencies (%), while continuous variables were reported as means (standard deviation [SD]) or medians (interquartile range [IQR]), as appropriate. Comparisons between groups were conducted using chi‐square or Fisher's exact tests for categorical variables and *t*‐tests or Mann–Whitney *U* tests for continuous variables.

EQ‐5D‐5L states were converted into health utility values using the corresponding country‐specific value sets. In the absence of pre‐illness HRQoL measurements in our study population, the impact of RSV infection was estimated by comparing utility values against country‐ and age‐specific population normative values [21, 23]. Lower EQ‐5D‐5L index values compared with population norms reflect reduced self‐reported HRQoL, indicating loss in quality of life. Additional details are provided in the **Table** **S1**.

In secondary analyses, we compared the clinical disease burden outcomes between RSV and influenza patients, excluding those co‐infected with both RSV and influenza. While baseline comparisons included all patients, follow‐up outcomes were limited to Dutch patients as influenza follow‐up data were available only for the Netherlands.

Missing data were not imputed. Participants who did not complete any follow‐up questionnaires (Days 14 and 30; *n* = 3/91, 3%) were excluded from healthcare resource use and cost analyses. Among those with missing data at Day 30 only (*n* = 6/91, 7%), we conservatively assumed that no further healthcare resource use beyond Day 14 occurred, based on the hypothesis that participants whose RSV infection resolved by Day 14 were less likely to complete the 30‐day follow‐up.

#### 2.3.2 Socioeconomic Burden

Costs were assessed from a healthcare system (direct costs) and societal perspective (direct and indirect costs) [24]. Direct costs per RSV episode were obtained by multiplying healthcare and medication utilization data by unit costs for each resource type (Table S2). Hospitalization costs were not included in the analysis, due to the low number of hospitalizations (*n* = 2) and limited available data for these patients. Indirect costs were obtained by multiplying workdays lost by salary unit costs. Workdays lost were defined as the sum of missed workdays of the patient itself and, possibly, any caretaker, due to the illness of the participant.

Unit costs for healthcare visits and medications were based on national prices, and salary unit costs were based on country‐specific average gross daily wages [25] inflated to 2023 Euros (€) [26]. A lognormal distribution was used to address non‐normally distributed parameters. We calculated 95% confidence intervals for cost estimates using bootstrapping with 10,000 samples. Further details are available in the Supporting Information.

#### 2.3.3 Estimation of the Primary Care RSV ARI Incidence

As an exploratory analysis, we estimated primary care RSV ARI incidence among older adults in the Dutch cohort. Because our study was neither designed nor powered to determine these estimates directly, we used pseudonymized routine care data from the Julius General Practitioners' Network (JGPN) database, which contains primary healthcare data for over 370,000 individuals in the Netherlands (Utrecht area) [27]. All Dutch practices who participated in the prospective cohort study are located within the catchment area of JGPN with the latter having a patient population representative for the Dutch population as a whole [27]. Some study practices are also part of the JGPN network, but data are anonymized and cannot be directly linked to our cohort. ARI episodes were identified by specific ICPC‐1 codes (see Supporting Information) registered during the study periods (November to March, 2022–2024).

Overall ARI incidence was calculated by dividing the total number of episodes by the total follow‐up time of individuals aged ≥ 60 years registered at JGPN practices. Because we did not have approval to access total ARI data directly from study practices, the RSV positivity rates observed in our Dutch study cohort were applied to the JGPN ARI incidence to estimate primary care RSV ARI incidence. This approach allows for population‐representative estimates without directly linking individual study practices to JGPN data. The monthly RSV positivity rates in the Dutch cohort of our study were then applied to these data to estimate primary care RSV ARI incidence. Incidence rates were reported as the number of episodes per 1000 person‐years. Exact 95% confidence intervals (95%CI) were derived using the Poisson distribution. In a sensitivity analysis, we evaluated the impact of extending our ARI definition by also including COPD and asthma exacerbations (see Supporting Information).

Statistical analyses were conducted using the SPSS (version 29) and R statistical software (version 4.3.1). JGPN data were managed using the myDRE platform.

## 3 Results

### 3.1 Study Population

**FIGURE 1:**
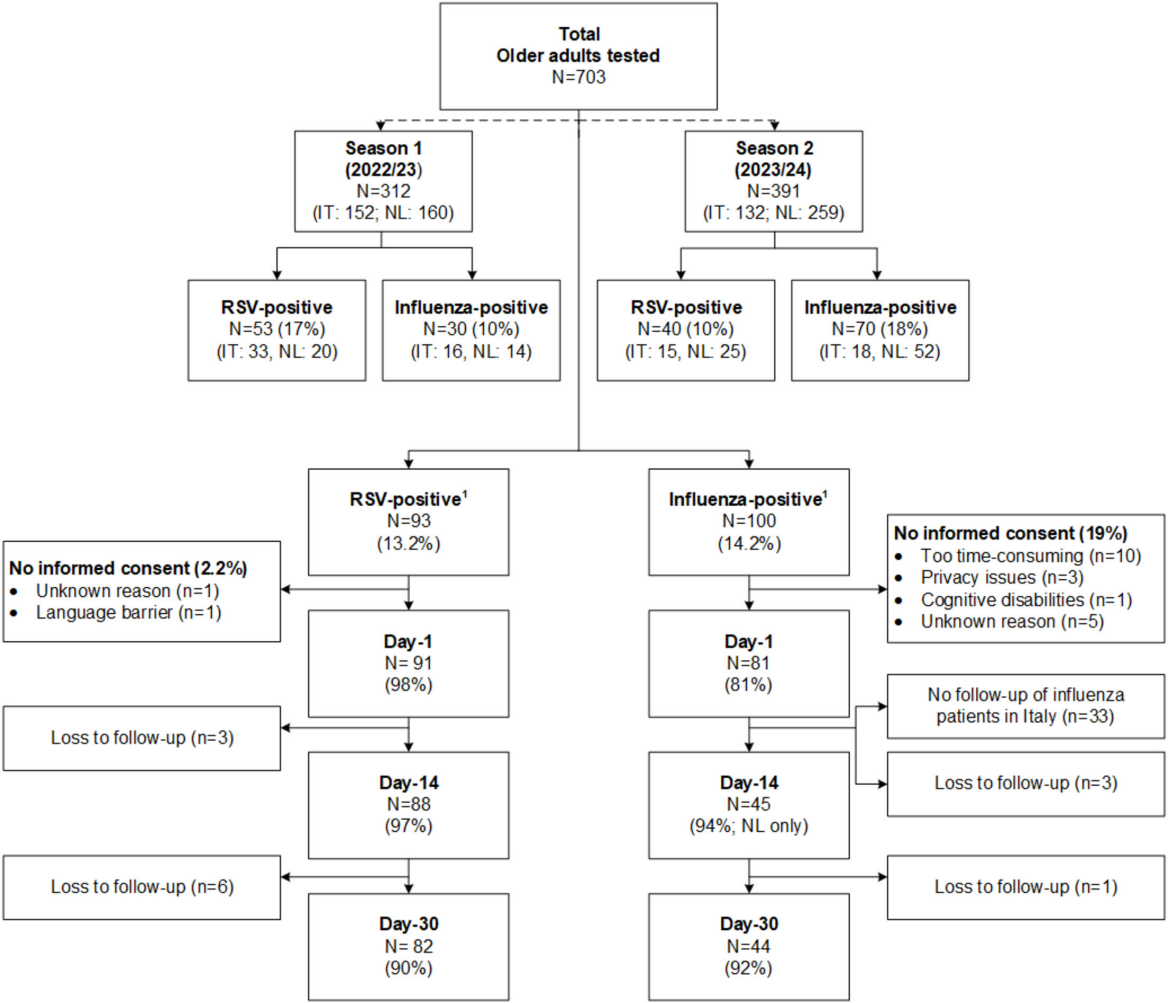
Flowchart. ^1^Two participants tested positive for both influenza and RSV.

Of the 703 older adults with ARI, 93 were positive for RSV (13.2%) and 100 for influenza (14.2%), with two positive for both RSV and influenza (0.3%) (Figure S1). RSV‐positivity rate ranged between 10% and 17% across seasons and 11% and 13% across countries. Figure 1 shows the rates stratified by both season and country. Day‐1 data were collected for 91/93 (98%) RSV patients, of which 88 (97%) completed the Day‐14 follow‐up questionnaire.

Mean age of RSV patients was 76 years (SD: 8), with 58% being female (Table 1). Most patients (63%) had at least one comorbidity, with cardiovascular and respiratory diseases being most frequently reported (25% and 23%, respectively). Compared with influenza patients, RSV patients were significantly older (76 years vs. 71 years, *p* < 0.001). Demographic and patient characteristics did not significantly differ between RSV patients from Italy and the Netherlands, except for employment status, with Dutch patients more frequently being employed (24% vs. 9%, *p* = 0.03) (Table S3).

**TABLE 1:** Baseline characteristics of RSV‐positive older adults.

	RSV‐positive^a^ *N* = 91	Influenza‐positive*N* = 79	*p*
Country			0.21
Italy	48 (52.7%)	34 (43.0%)	—
The Netherlands	43 (47.3%)	45 (57.0%)	—
Age, y, %			<0.001
60–69	19 (20.9%)	37 (46.8%)	—
70–79	44 (48.4%)	31 (39.2%)	—
80+	28 (30.8%)	11 (13.9%)	—
Age, y, mean	76.3 (8.0)	71.2 (8.5)	<0.001
Female sex	53 (58.2%)	48 (60.8%)	0.74
Chronic disease, any^b^	57 (62.6%)	39/60 (65.0%)	0.77
Respiratory disease	21 (23.1%)	16/60 (26.7%)	0.62
Asthma	6 (6.6%)	10/60 (16.7%)	0.05
COPD	12 (13.2%)	7/60 (11.7%)	0.78
Cardiovascular disease	23 (25.3%)	9/60 (15.0%)	0.13
Neurological disease	3 (3.3%)	0/60 (0.0%)	0.28
Rheumatic disease	4 (5.3%)	3/60 (5.0%)	1.00
Malignancy	2 (2.2%)	2/60 (3.3%)	0.65
Diabetes mellitus	11 (12.1%)	6/60 (10.0%)	0.69
Chronic medication use			
Prednisone use	1/58 (1.7%)	1/45 (2.2%)	1.00
Respiratory medicine	15/58 (25.9%)	14/45 (31.1%)	0.56
Smoking			0.48
Former	30/89 (33.7%)	26/60 (43.3%)	
Current	6/89 (6.7%)	3/60 (5.0%)	
Employment			
Currently employed	14/86 (16.3%)	16/45 (35.6%)	0.04
Vaccination status			
Influenza vaccine^c^	68/84 (81.0%)	44/55 (80.0%)	0.89
COVID vaccine^d^	80/90 (88.9%)	49/60 (81.7%)	0.25
Pneumococcal vaccine	38/70 (54.3%)	30/51 (58.8%)	0.62
Viral codetection^e^	10/48 (20.8%)	3/34 (8.8%)	0.14
Symptoms at presentation			
Dyspnea	31/88 (35.2%)	31/79 (39.2%)	0.62
Wheezing	23/56 (41.1%)	21/64 (32.8%)	0.39
Coughing, any	86/88 (97.7%)	77/79 (97.5%)	1.00
Productive cough	53/88 (60.2%)	48/77 (62.3%)	0.87
Sore throat	34/88 (38.6%)	27/79 (34.2%)	0.44
Coryza	75/88 (85.2%)	57/79 (72.2%)	0.03
Fever ≥ 38°C	27/88 (30.7%)	45/79 (57.0%)	<0.001
Muscle pain	20/56 (35.7%)	46/64 (71.9%)	<0.001
Headache	22/56 (39.3%)	35/64 (54.7%)	0.08
Disturbed sleep	27/41 (65.9%)	34/45 (75.6%)	0.36
Fatigue	37/69 (53.6%)	57/69 (82.6%)	<0.001
Loss of appetite	14/69 (20.3%)	42/69 (60.9%)	<0.001

Abbreviations: COPD, chronic obstructive pulmonary disease.

^a^ For this baseline comparison, patients co‐infected with both RSV and influenza (*n* = 2) are included in the RSV‐positive group.

^b^ Cardio‐vascular diseases included heart failure, angina, infarction, arrhythmias, and structural heart diseases; neurological disease included Parkinson's disease, epilepsy, multiple sclerosis, Alzheimer's disease. Rheumatic disease included arthritis, rheuma, Bechterew, polymyalgia rheumatica, and fibromyalgia. Malignancy included active malignant conditions, excluding nonmetastatic skin cancer; diabetes mellitus was defined as Type 1, Type 2, or unspecified diabetes. Hypertension was not considered a chronic disease.

^c^ Influenza vaccination in the past 6 months.

^d^ At least one COVID‐vaccination.

^e^ Among 10 RSV‐positive patients with viral codetection: six had hMPV, one had influenza, one had rhinovirus, one had bocavirus, and one had parainfluenza virus. These data were available only for patients from Italy, which were tested by multiplex RT‐PCR. Among influenza‐positive patients, one had rhino and two had SARS‐CoV‐2. Patients from the Netherlands were tested solely for RSV and influenza using point‐of‐care diagnostics (Abbott ID NOW), which identified one additional RSV‐influenza codetection.

### 3.2 Clinical Disease Burden

**FIGURE 2:**
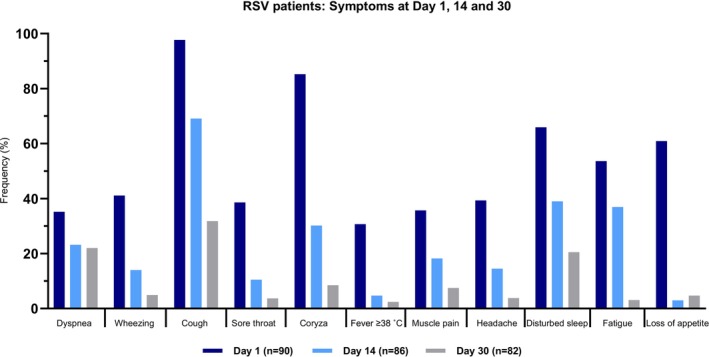
Symptoms of RSV‐positive patients at Days 1, 14, and 30 after the initial primary care visit.

Figure 2 presents symptoms of RSV patients on Days 1, 14, and 30. Cough was most frequently reported at presentation (Day 1, 98%) and remained the most frequently reported symptom during follow‐up (Day 30, 32%). On average, RSV illness lasted for 17 days (SD: 10).

During 30 days of follow‐up, 38% (33/87) of RSV patients had at least one repeat primary care visit (i.e., ≥ 2 consultations for the same illness), 5% (4/88) were referred to the emergency department, and 2% (2/88) were hospitalized (Table 2). Prescribed medication use was reported in 61% of RSV episodes, with antibiotics (40%) and systemic steroids (21%) being most frequently prescribed. Over‐the‐counter medication use was reported in 54%.

**TABLE 2:** Healthcare and medication use of RSV‐positive older adults.

Healthcare use	
≥ 1 repeat GP visit^a^, ^c^	33/87 (37.9%)
Visit to GP	29/87 (33.3%)
Home visit by GP	8/87 (9.2%)
Out‐of‐hours GP visit	2/86 (2.3%)
Number GP visits^b^, ^c^	
Mean (SD)	1.7 (1.3)
Median (IQR)	1.0 (1.0–2.0)
Emergency department visit	4/88 (4.5%)
Hospitalization^d^	2/88 (2.3%)
Medication use	
Medication use, any	76/87 (87.4%)
Prescribed medication, any	53/87 (60.9%)
Antibiotics	35/87 (40.2%)
Respiratory inhalers	
Beta‐2‐sympatics	16/87 (18.4%)
Steroid	14/87 (16.1%)
Systemic steroids	18/87 (20.7%)
Over‐the‐counter, any	47/87 (54.0%)
Antipyretic or pain medication	33/86 (38.4%)
Nasal spray	11/87 (12.6%)
Cough syrup or tablets	24/86 (27.9%)

Abbreviations: GP, general practitioner.

^a^ At least one visit after the initial GP visit related to this ARI episode.

^b^ All GP visits (visit to GP, home visits by GP, and out‐of‐hours GP visits), including the initial visit.

^c^ GP visits were defined as physical visits to, or home visits by, the GP, including out‐of‐hours GP consultations, related to this episode of ARI.

^d^ Hospitalization was defined as hospital admission due to the RSV infection for at least 1 day (24 h).

**FIGURE 3:**
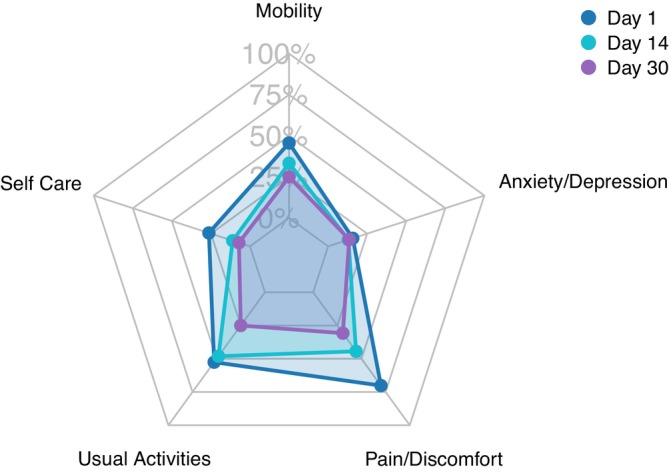
Proportion of RSV‐positive patients reporting any health‐related quality of life problems in each EQ‐5D‐5L domain, at Days, 14, and 30 after the initial primary care visit.

Figure 3 shows the proportion of RSV patients reporting any problems in HRQoL domains at Days 1, 14, and 30. At Day 1, RSV patients most often reported problems regarding discomfort/pain (70%) and daily activities (53%) (Table S5). Compared with age‐specific population norms, EQ‐5D mean utility values were significantly lower at Day 1 (−0.11, *p <* 0.001*)* but not at Day 14 (−0.02, *p =* 0.43) and Day 30 (+0.03, *p =* 0.13).

RSV patients from Italy more often reported repeat primary care visits compared with those from the Netherlands (51% vs. 24%, *p =* 0.009) and were prescribed systemic steroids more frequently (31% vs. 10%, *p =* 0.02) (Table S10), whereas Dutch patients reported a longer mean illness duration (19.6 vs. 11.8 days, *p* < 0.001) (Table S8). Mean EQ‐5D utility value at Day 1 was lower in the Netherlands compared with Italy, but not at other timepoints (Table S5). Among RSV patients, clinical burden outcomes did not significantly differ between those aged 60–74 and ≥ 75 years (**Tables** **S4**, **S9**, **S11**), with the exception of more frequently reported mobility problems on Days 1 and 14 in the ≥ 75 age group (Table S6).

Compared with influenza patients, RSV patients had significantly less fever, muscle pain, loss of appetite, and fatigue at presentation (Day 1) (Table 1). Healthcare visits, medication use, and EQ‐5D utility values did not significantly differ between RSV and influenza patients (Tables S7 and S12).

### 3.3 Socioeconomic Burden

Work absence due to RSV illness was reported by 9% (8/85) of all RSV patients (57% among those employed), and 6% (4/67) indicated that a partner, family member of other caretaker had to take days off to provide care. Reported work absence did neither significantly differ between countries (Table S13), nor between RSV and influenza patients (Table S15). Work absence was more frequently reported in RSV patients aged 60–74 years compared with those aged ≥ 75 years (18.9% vs. 2.1%, *p* = 0.019; Table S14).

Overall, the mean costs per RSV episode were estimated at €78.1 (95%CI: 74.4–81.8) from a healthcare system perspective, and €279.7 (95%CI: 245.5–318.2) from a societal perspective. Costs per RSV episode stratified by country and age are provided in Table 3 and Table S16, respectively. Mean healthcare system costs per RSV episode were similar between the Netherlands and Italy (€78.0 [95%CI: 73.8–82.2] vs. €70.5; 95%CI: 67.6–73.5), whereas societal costs were higher in the Netherlands (€408.2 [95%CI: 359.2–463.4] vs. €166.6 (95%CI: 147.2–188.5). Mean healthcare system costs were higher in RSV patients aged ≥ 75 years (€85.2 [95%CI: 81.4–89.3] vs. €69.5 [95%CI: 66.0–73.2]), while societal costs were higher in those aged 60–75 years (€408.1 [95%CI: 361.7–459.9] vs. €182.7 [95%CI: 160.9–207.5]). Compared with influenza, costs per RSV episode did not significantly differ for both perspectives (Table S17).

**TABLE 3:** Costs per RSV episode.

	Total	The Netherlands	Italy
Healthcare system costs^a^			
Mean (95% CI)	€78.07 (74.37–81.84)	€77.95 (73.81–82.17)	€70.53 (67.58–73.52)
Median (IQR)	€47.11 (34.66–73.68)	€44.17 (33.82–66.11)	€46.67 (30.81–79.43)
Societal costs^b^			
Mean (95% CI)	€279.73 (245.52–318.23)	€408.24 (359.20–463.40)	€166.62 (147.17–188.53)
Median (IQR)	€50.93 (34.66–94.94)	€48.13 (33.82–100.85)	€51.11 (30.81–97.45)

*Note:* Confidence intervals of means were calculated by using bootstrapping (10,000 bootstrap samples). Country‐specific cost calculations were based on country‐specific healthcare use and national unit prices, adjusted for PPP.

^a^ Healthcare system costs do not include hospitalization costs.

^b^ Societal costs include productivity losses resulting from workdays lost by the patient as well as by a caretaker. Higher mean societal costs in the Netherlands compared with Italy are explained by more frequent work absence (Table S13) and higher unit costs of work absence (Supplementary Methods). As work absence was reported only by a minority of patients (8/85), productivity losses had little impact on median societal costs.

### 3.4 Primary Care RSV ARI Incidence

The estimated annual primary care ARI incidence rate in the Netherlands was 251 episodes per 1000 person‐years (95% CI: 234–267) in 2022–2023, and 190 per 1000 person‐years (95% CI: 166–215) in 2023–2024 (Table S18). The primary care RSV ARI incidence was estimated at 11 episodes per 1000 person‐years (95% CI: 8–16) in 2022–2023 and eight per 1000 person‐years (95% CI: 4–15) in 2023–2024. As such, RSV accounts for 4.2%–4.5% of older adult ARI episodes in primary care year‐round.

A sensitivity analysis showed that the overall RSV ARI incidence rate was 1.5‐fold higher (15 vs. 10 episodes per 1000 persons‐years) when COPD and asthma exacerbations were also included in the ARI episode definition (Table S18).

## 4 Discussion

In this prospective primary‐care based cohort study during two consecutive RSV seasons in two European countries, RSV was detected in 13% of older adults presenting with ARI. Albeit no RSV‐related deaths occurred and only a few cases required ED visits (5%; 4/88) or hospitalization (2%; 2/88), RSV infections were associated with considerable illness duration, symptomatology, healthcare and medication utilization, and costs, comparable with those caused by influenza.

Our RSV‐positivity rate aligns with prospective studies from the United States and Greece, reporting 10%–11% of older adults with medically attended ARI testing positive for RSV during winter [5, 6]. A Europe‐wide primary care study, however, found lower RSV‐positivity rates (5.2% in those 60–74 years and 8.5% in those 75+), but tested year‐round, hampering direct comparison [8]. Although those applying the ILI rather than the ARI case definition are also not directly comparable [13], such studies identified RSV in 7.4% (moderate‐to‐severe ILI) and 15% (all‐type ILI) of older adults [11, 12]. Our estimation of annual RSV ARI incidence in primary care of 10 per 1000 person‐years (range: 8–11 across seasons) is comparable with studies in Europe and the United States, reporting 0.5%–1.4% of older adults attending outpatient care with RSV ARI annually [2, 4, 6].

While RSV infections requiring hospitalization are associated with significant morbidity and mortality [28, 29, 30], community studies suggest RSV infections in community‐dwelling older adults are typically mild [2, 4]. Korsten et al. and Falsey et al. reported no hospitalizations or deaths out of 36 community‐dwelling and 46 healthy older adults with RSV infection [2, 4], respectively. Similarly, Bruyndonckx et al. observed only one hospitalization in 122 RSV‐infected adults aged ≥ 18 years seeking primary care [8]. However, in adults with cardiopulmonary comorbidities, Falsey et al. found that 16% were hospitalized, and 4% died [4], indicating a higher risk of severe outcomes for those with significant underlying medical conditions.

Despite the low hospitalization rate in our study, outpatient RSV infections in older adults still posed a considerable clinical and socioeconomic burden. Given that medically attended outpatient RSV is far more common than RSV‐associated hospitalization, the cumulative impact on older adults and the healthcare system remains substantial. Consistent with prior studies [2, 4, 6, 31], we observed frequent primary care repeat visits and prescriptions (especially antibiotics and steroids), and illness generally lasting for more than 2 weeks. Similar to previous studies [10, 31], we observed a temporary decline in quality of life, with EQ‐5D utility values dropping during the first week of illness and recovering within 3 to 4 weeks.

Only one study has reported on the economic burden of outpatient RSV infections based on data directly derived from older adults [10]. This study, based on only 11 medically attended RSV cases across three European countries, estimated direct costs per episode at €75.2 (median, €65.3) from a healthcare payers' perspective, comparable with our finding of €78.1 (median, €47.1). No work absence was observed, nor did they include productivity losses due to informal caregiving. However, in our study, these indirect costs were substantial, especially among 60–74‐year‐olds who were relatively frequently employed. In a German health claim database study, healthcare costs per outpatient RSV episode ranged from €106 in adults aged 60–74 years to €200 in those aged ≥ 85 years, although the lack of a detailed costs breakdown limits comparability. Nonetheless, the authors concluded that outpatient cases accounted for 25% of the overall economic RSV burden in Germany [32].

The burden of RSV in older adults is often compared with influenza [1]. Previous studies suggest that, while influenza may be more prevalent in outpatient settings [2, 6, 8, 11], disease severity is comparable [2, 11]. Our findings also show similar illness duration, healthcare resource use, and costs for RSV and influenza infections. However, comparisons between RSV and influenza should be interpreted with caution; high influenza vaccination coverage in our study population—and likely in the referenced studies—may have mitigated the incidence and severity of symptomatic influenza among those vaccinated.

Our study has several important strengths. The sample size of our observational study is sufficiently large to accurately capture the burden of outpatient RSV infections among older adults. Its prospective design with comprehensive follow‐up for 30 days allowed us to capture both clinical and socioeconomic impacts of RSV infection. With enrollment spanning two seasons across two European countries, this study represents the largest outpatient cohort of RSV‐infected older adults in Europe to date.

Several limitations should be acknowledged. Most importantly, selection bias may have influenced our findings. Patients who declined participation may differ from those who agreed, though the lack of data among those having declined participations preclude us from assessing possible selection bias. Also, we may have missed patients with severe ARI who deteriorated too quickly for a swab to be collected or who sought care at out‐of‐hours GP or ED services, potentially underrepresenting the most severe cases. Second, differences in RSV‐positivity rates between Italy and Netherlands may indicate local and seasonal epidemiological variation, but could also reflect differences in diagnostic methods. Although we lack data on diagnostic performance of the point‐of‐care test (Abbott ID NOW) in older adults, this device showed high accuracy compared with RT‐PCR in children [33]. Third, our study relied on nasopharyngeal samples for RSV detection, as did all aforementioned observational studies [2, 4, 6, 8, 9, 11], which have limited sensitivity for detecting RSV. Consequently, the true burden of RSV is likely substantially underestimated; detection rates may be up to 1.5–2.2 times higher when adding serology and/or sputum samples [34, 35]. Fourth, RSV ARI primary care incidence estimates were based on data solely from the Netherlands, limiting generalizability to other European countries with different RSV epidemiology or healthcare‐seeking behaviors. Fifth, comparison of course of illness between RSV and influenza were restricted to the Netherlands, as influenza patients were not followed up after Day 1 in Italy. Sixth, pre‐illness EQ‐5D‐5L values were not available for our population. We therefore compared HRQoL during RSV episodes with country‐specific population norms for older adults, which may have slightly overestimated the decline in HRQoL as our study population could potentially have had lower baseline health status than the general older adult population. Seventh, in cost analysis, we were unable to take certain healthcare expenses into account, such as remote consultations and diagnostic tests (e.g., X‐rays), resulting in conservative cost estimates. Finally, coinfections with other viral pathogens were identified in 21% (10/48) of those tested with a full respiratory panel, with the majority being hMPV‐RSV coinfections (6/10). In these cases, we cannot definitively attribute ARI and associated disease burden exclusively to RSV. Nonetheless, RSV detection in adults with ARI symptoms has been strongly associated with the presence of respiratory symptoms [36]. Furthermore, while viral coinfections have been linked to more severe outcomes in hospitalized older patients with RSV [37, 38], to our knowledge, comparable data for outpatient settings are lacking, and the specific contribution of multiple pathogens to ARI severity remains unclear. Given the small number of coinfected cases, we were unable to further explore the role of coinfections in RSV disease severity, warranting further research.

Our findings are particularly timely given the recent approval of RSV vaccines for older adults, which have shown promising efficacy in preventing severe RSV infections [15, 16, 17]. Some countries have incorporated these vaccines into immunization programs, with recommendations regarding age thresholds and indications varying widely [18], whereas other countries are still deliberating their introduction. The data presented here contribute to a more complete understanding of the overall burden of RSV, which is essential for informing public health planning and policy decisions. Additionally, our findings could help raise awareness and improve current knowledge of RSV among both (primary) healthcare providers and older adults [39, 40].

Altogether, our study sheds light on the clinical and socioeconomic burden of RSV in older adults seeking primary care in Europe. Although hospitalization is infrequent, the primary care burden of RSV infections in older adults is considerable.

## Author Contributions

The corresponding author attests that all listed authors meet authorship criteria, that no others meeting the criteria have been omitted, and that all authors have seen and approved of the final text. **Sarah F. Hak, Joanne G. Wildenbeest, Louis J. Bont, Caterina Rizzo, Roderick P. Venekamp**: conceptualization and methodology. **Sarah F. Hak, Sara Bracaloni, Emma te Bogt, Eugenio Mammolenti, Michela Scarpaci, Joanne G. Wildenbeest, Leonardo Bonaldo, Caterina Rizzo, RPV**: data curation and analysis. **Jacqueline Vlaskamp‐Smit, Sarah F. Hak, Sara Bracaloni, Michela Scarpaci, Tommaso Cosci, Eugenio Mammolenti, Melissa Torrisi, Leonardo Bonaldo, Enrica Esposito, Maria Chironna, Donatella Panatto, Giancarlo Icardi**: investigation. **Joanne G. Wildenbeest, Louis J. Bont, Caterina Rizzo, Roderick P. Venekamp**: supervision. **Sarah F. Hak**: visualization. **Sarah F. Hak, Emma te Bogt, Eugenio Mammolenti, Melissa Torrisi, Joanne G. Wildenbeest, Louis J. Bont, Caterina Rizzo, Roderick P. Venekamp**: writing – original draft. **All authors**: writing – review and editing.

## Conflicts of Interest

EB, EE, EM, GI, JV, LB, MC, MS, MT, RPV, SB, SH, TC, and DP report no potential conflict of interest. CR declares that she received fees for participation in advisory boards from AstraZeneca, Seqirus, MSD, Sanofi, and GSK and for CME lectures from Seqirus, Sanofi, AstraZeneca, MSD, and GSK. JW has been an investigator for clinical trials sponsored by pharmaceutical companies including AstraZeneca, Merck, Pfizer, Sanofi, and Janssen. All funds have been paid to UMCU. JW participated in the advisory board of Janssen and Sanofi with fees paid to UMCU. LJB has regular interaction with pharmaceutical and other industrial partners. He has not received personal fees or other personal benefits. UMCU has received major funding (> €100,000 per industrial partner) for investigator‐initiated studies from AbbVie, MedImmune, AstraZeneca, Sanofi, Janssen, Pfizer, MSD, and MeMed Diagnostics. UMCU has received major funding for the RSV GOLD study from the Bill & Melinda Gates Foundation. UMCU has received major funding as part of the public–private partnership IMI‐funded RESCEU and PROMISE projects with partners GSK, Novavax, Janssen, AstraZeneca, Pfizer, and Sanofi. UMCU has received major funding from Julius Clinical for participating in clinical studies sponsored by MedImmune and Pfizer. UMCU received minor funding (€1000–25,000 per industrial partner) for consultation and invited lectures by AbbVie, MedImmune, Ablynx, Bavaria Nordic, MabXience, GSK, Novavax, Pfizer, Moderna, AstraZeneca, MSD, Sanofi, Genzyme, and Janssen. LJB is the founding chairman of the ReSViNET Foundation. CR participated in the Advisory Board and Expert scientific discussion for Seqirus, MSD, GSK, Sanofi, and AstraZeneca.

## Peer Review

The peer review history for this article is available at https://www.webofscience.com/api/gateway/wos/peer‐review/10.1111/irv.70174.

## Ethics Statement and Consent

Ethical approval was obtained from the medical ethics committees in Italy by the Ethical Committee “Comitato Etico di Area Vasta Nord Ovest (CEAVNO) per la Sperimentazione clinica” of the Tuscany Region (ref 22871_Dini). In the Netherlands, a waiver for official ethical approval was granted by the Medisch Ethische Toetsingscommissie Utrecht (ref 22‐667), indicating that the medical research ethics committee decided that this research does not fall under the scope of the Dutch Medical Research Involving Human Subjects Act, and therefore, official ethical approval was not required. All participants provided written or electronic informed consent.

## Supporting information


**Table S1:** Population norms for Italy [15, 16] and The Netherlands [14].
**Table S2:** Unit costs.
**Table S3:** Patient characteristics of RSV patients by country.
**Table S4:** Patient characteristics of RSV patients by age.
**Table S5:** HRQoL in RSV patients overall and by country.
**Table S6:** HRQoL in RSV patients by age.
**Table S7:** HRQoL in RSV versus influenza patients.
**Table S8:** Disease characteristics of RSV patients by country.
**Table S9:** Disease characteristics of RSV patients by age.
**Table S10:** Healthcare resource use in RSV patients by country.
**Table S11:** Healthcare resource use in RSV patients by age.
**Table S12:** Healthcare resource use RSV versus influenza (NL data only).
**Table S13:** Work absenteeism in RSV patients overall and by country.
**Table S14:** Work absenteeism in RSV patients by age.
**Table S15:** Work absenteeism in RSV‐positive versus influenza (NL data only).
**Table S16:** Costs of RSV episodes by age.
**Table S17:** Costs of RSV and influenza episode (NL data only).
**Table S18:** Incidence of RSV infections in primary care among older adults (NL only).
**Figure S1:** Number of RSV and influenza positive swabs.

## Data Availability

Upon publication of this manuscript, anonymized data are available on reasonable request. Inquiries can be sent to the corresponding author. Data from the Julius General Practitioners Network (JGPN), used to estimate incidence, cannot be made publicly available due to ethical and legal restrictions. However, all interested readers may request data without restriction from the JGPN board (secretariaatjhn-3@umcutrecht.nl).

## References

[1] J. G. Wildenbeest , D. M. Lowe , J. F. Standing , and C. C. Butler , “Respiratory Syncytial Virus Infections in Adults: A Narrative Review,” Lancet Respiratory Medicine Published online 12 (2024): 822–836, 10.1016/S2213-2600(24)00255-8. 39265602

[2] K. Korsten , N. Adriaenssens , S. Coenen , et al., “Burden of Respiratory Syncytial Virus Infection in Community‐Dwelling Older Adults in Europe (RESCEU): An International Prospective Cohort Study,” European Respiratory Journal 57, no. 4 (2021): 2002688, 10.1183/13993003.02688-2020. 33060153

[3] K. G. Nicholson , J. Kent , V. Hammersley , and E. Cancio , “Acute Viral Infections of Upper Respiratory Tract in Elderly People Living in the Community: Comparative, Prospective, Population Based Study of Disease Burden,” British Medical Journal Published online 315 (1997): 1060–1064, 10.1136/bmj.315.7115.1060. PMC2127683 9366736

[4] A. R. Falsey , P. A. Hennessey , M. A. Formica , C. Cox , E. E. Walsh , “Respiratory Syncytial Virus Infection in Elderly and High‐Risk Adults,” Published online (2005), www.nejm.org. 10.1056/NEJMoa043951 15858184

[5] E. Antalis , Z. Oikonomopoulou , C. Kottaridi , et al., “Mixed Viral Infections of the Respiratory Tract: An Epidemiological Study During Consecutive Winter Seasons,” Journal of Medical Virology 90, no. 4 (2018): 663–670, 10.1002/jmv.25006. 29244214 PMC7167177

[6] E. A. Belongia , J. P. King , B. A. Kieke , et al., “Clinical Features, Severity, and Incidence of RSV Illness During 12 Consecutive Seasons in a Community Cohort of Adults ≥60 Years Old. *Open Forum* ,” Infectious Diseases 5, no. 12 (2018): ofy316, 10.1093/ofid/ofy316. PMC6306566 30619907

[7] M. Savic , Y. Penders , T. Shi , A. Branche , and J. Y. Pirçon , “Respiratory Syncytial Virus Disease Burden in Adults Aged 60 Years and Older in High‐Income Countries: A Systematic Literature Review and Meta‐Analysis,” Influenza and Other Respiratory Viruses 17, no. 1 (2023): e13031, 10.1111/irv.13031. 36369772 PMC9835463

[8] R. Bruyndonckx , S. Coenen , C. Butler , et al., “Respiratory Syncytial Virus and Influenza Virus Infection in Adult Primary Care Patients: Association of Age With Prevalence, Diagnostic Features and Illness Course,” International Journal of Infectious Diseases 95 (2020): 384–390, 10.1016/j.ijid.2020.04.020. 32320810 PMC7167228

[9] M. E. Sundaram , J. K. Meece , F. Sifakis , R. A. Gasser , and E. A. Belongia , “Medically Attended Respiratory Syncytial Virus Infections in Adults Aged ≥50 Years: Clinical Characteristics and Outcomes,” Clinical Infectious Diseases 58, no. 3 (2014): 342–349, 10.1093/cid/cit767. 24265361 PMC7108027

[10] Z. Mao , X. Li , K. Korsten , et al., “Economic Burden and Health‐Related Quality of Life of Respiratory Syncytial Virus and Influenza Infection in European Community‐Dwelling Older Adults,” Journal of Infectious Diseases 226 (2022): S87–S94, 10.1093/infdis/jiac069. 35961055

[11] A. R. Falsey , J. E. McElhaney , J. Beran , et al., “Respiratory Syncytial Virus and Other Respiratory Viral Infections in Older Adults With Moderate to Severe Influenza‐Like Illness,” Journal of Infectious Diseases 209, no. 12 (2014): 1873–1881, 10.1093/infdis/jit839. 24482398 PMC4038137

[12] M. C. Zambon , J. D. Stockton , J. P. Clewley , and D. M. Fleming , “Contribution of Influenza and Respiratory Syncytial Virus to Community Cases of Influenza‐Like Illness: An Observational Study,” Lancet 358, no. 9291 (2001): 1410–1416, 10.1016/S0140-6736(01)06528-X. 11705487

[13] K. Korsten , N. Adriaenssens , S. Coenen , et al., “World Health Organization Influenza‐Like Illness Underestimates the Burden of Respiratory Syncytial Virus Infection in Community‐Dwelling Older Adults,” Journal of Infectious Diseases 226 (2022): S71–S78, 10.1093/infdis/jiab452. 34904176 PMC9374507

[14] R. T. Stein and H. J. Zar , “RSV Through the COVID‐19 Pandemic: Burden, Shifting Epidemiology, and Implications for the Future,” Pediatric Pulmonology 58, no. 6 (2023): 1631–1639, 10.1002/ppul.26370. 36811330

[15] E. E. Walsh , G. Pérez Marc , A. M. Zareba , et al., “Efficacy and Safety of a Bivalent RSV Prefusion F Vaccine in Older Adults,” New England Journal of Medicine 388, no. 16 (2023): 1465–1477, 10.1056/nejmoa2213836. 37018468

[16] A. Papi , M. G. Ison , J. M. Langley , et al., “Respiratory Syncytial Virus Prefusion F Protein Vaccine in Older Adults,” New England Journal of Medicine 388, no. 7 (2023): 595–608, 10.1056/nejmoa2209604. 36791160

[17] E. Wilson , J. Goswami , A. H. Baqui , et al., “Efficacy and Safety of an mRNA‐Based RSV PreF Vaccine in Older Adults,” New England Journal of Medicine 389, no. 24 (2023): 2233–2244, 10.1056/nejmoa2307079. 38091530

[18] J. Terstappen , S. F. Hak , A. Bhan , et al., “The Respiratory Syncytial Virus Vaccine and Monoclonal Antibody Landscape: The Road to Global Access,” Lancet Infectious Diseases Published online 24 (2024): e747–e761, 10.1016/S1473-3099(24)00455-9. PMC12311909 39326422

[19] World Health Organization (WHO) , “RSV Surveillance Case Definitions,” retrieved August 20, 2025, https://www.who.int/teams/global‐influenza‐programme/global‐respiratory‐syncytial‐virus‐surveillance/case‐definitions.

[20] A. P. Finch , M. Meregaglia , O. Ciani , B. Roudijk , and C. Jommi , “An EQ‐5D‐5L Value Set for Italy Using Videoconferencing Interviews and Feasibility of a New Mode of Administration,” Social Science & Medicine 292 (2022): 292, 10.1016/j.socscimed.2021.114519. 34736804

[21] M. Versteegh , K. M. Vermeulen , S. M. A. A. Evers , G. A. de Wit , R. Prenger , and E. A. Stolk , “Dutch Tariff for the Five‐Level Version of EQ‐5D,” Value in Health 19, no. 4 (2016): 343–352, 10.1016/j.jval.2016.01.003. 27325326

[22] EuroQol Research Foundation , “EQ‐5D User Guides 2020,” retrieved July 6, 2023, https://euroqol.org/publications/user‐guides.

[23] M. Meregaglia , F. Malandrini , A. P. Finch , O. Ciani , and C. Jommi , “EQ‐5D‐5L Population Norms for Italy,” Applied Health Economics and Health Policy 21, no. 2 (2023): 289–303, 10.1007/s40258-022-00772-7. 36434410 PMC9702834

[24] Y. Kim , Y. Kim , H. J. Lee , et al., “The Primary Process and Key Concepts of Economic Evaluation in Healthcare,” Journal of Preventive Medicine and Public Health 55, no. 5 (2022): 415–423, 10.3961/jpmph.22.195. 36229903 PMC9561137

[25] Eurostat , “Mean Annual Earnings by Sex, Age and Economic Activity,” retrieved October 17, 2024, https://ec.europa.eu/eurostat/databrowser/view/earn_ses18_27/default/table?lang=en&category=labour.earn.earn_ses2018.earn_ses18_an.

[26] Eurostat , “Harmonised Index of Consumer Prices (HICP) Annual Data,” retrieved October 17, 2024, https://ec.europa.eu/eurostat/databrowser/view/prc_hicp_aind/default/table?lang=en&category=prc.prc_hicp.

[27] H. M. Smeets , M. F. Kortekaas , F. H. Rutten , et al., “Routine Primary Care Data for Scientific Research, Quality of Care Programs and Educational Purposes: The Julius General Practitioners' Network (JGPN),” BMC Health Services Research 18, no. 1 (2018): 735, 10.1186/s12913-018-3528-5. 30253760 PMC6156960

[28] H. Celante , N. Oubaya , S. Fourati , et al., “Prognosis of Hospitalised Adult Patients With Respiratory Syncytial Virus Infection: A Multicentre Retrospective Cohort Study,” Clinical Microbiology and Infection 29, no. 7 (2023): 943.e1–943.e8, 10.1016/j.cmi.2023.03.003. 36914069

[29] N. Lee , G. C. Y. Lui , K. T. Wong , et al., “High Morbidity and Mortality in Adults Hospitalized for Respiratory Syncytial Virus Infections,” Clinical Infectious Diseases 57, no. 8 (2013): 1069–1077, 10.1093/cid/cit471. 23876395

[30] B. Ackerson , H. F. Tseng , L. S. Sy , et al., “Severe Morbidity and Mortality Associated With Respiratory Syncytial Virus Versus Influenza Infection in Hospitalized Older Adults,” Clinical Infectious Diseases 69, no. 2 (2019): 197–203, 10.1093/cid/ciy991. 30452608 PMC6603263

[31] H. Ohbayashi , T. Sakurai , D. Himeji , et al., “Burden of Respiratory Syncytial Virus Infections in Older Adults With Acute Respiratory Infection in Japan: An Epidemiological Study Among Outpatients,” Respiratory Investigation 62, no. 5 (2024): 914–921, 10.1016/j.resinv.2024.06.003. 39126825

[32] B. Huebbe , A. Mocek , K. C. Manz , et al., “Economic Burden of Respiratory Syncytial Virus in Adults in Germany—A Health Claims Analysis bBtween 2015 and 2018,” Journal of Medical Economics 27, no. 1 (2024): 1063–1075, 10.1080/13696998.2024.2389676. 39105626

[33] S. V. Schnee , J. Pfeil , C. M. Ihling , J. Tabatabai , and P. Schnitzler , “Performance of the Alere i RSV Assay for Point‐of‐Care Detection of Respiratory Syncytial Virus in Children,” BMC Infectious Diseases 17, no. 1 (2017): 767, 10.1186/s12879-017-2855-1. 29237419 PMC5729395

[34] C. Onwuchekwa , L. M. Moreo , S. Menon , et al., “Underascertainment of Respiratory Syncytial Virus Infection in Adults Due to Diagnostic Testing Limitations: A Systematic Literature Review and Meta‐Analysis,” Journal of Infectious Diseases Published online 228 (2023): 173–184, 10.1093/infdis/jiad012. PMC10345483 36661222

[35] Y. Li , D. Kulkarni , E. Begier , et al., “Adjusting for Case Under‐Ascertainment in Estimating RSV Hospitalisation Burden of Older Adults in High‐Income Countries: A Systematic Review and Modelling Study,” Infection and Drug Therapy Published online April 1 (2023): 1137–1149, 10.1007/s40121-023-00792-3. PMC10027261 36941483

[36] L. M. Vos , R. Bruyndonckx , N. P. A. Zuithoff , et al., “Lower Respiratory Tract Infection in the Community: Associations Between Viral Aetiology and Illness Course,” Clinical Microbiology and Infection 27, no. 1 (2021): 96–104, 10.1016/j.cmi.2020.03.023. 32244051 PMC7118666

[37] M. Haeberer , M. Mengel , R. Fan , et al., “RSV Risk Profile in Hospitalized Adults and Comparison With Influenza and COVID‐19 Controls in Valladolid, Spain, 2010–2022,” Infectious Disease and Therapy 13, no. 9 (2024): 1983–1999, 10.1007/s40121-024-01021-1. PMC11343947 39033476

[38] A. Ferrari , I. Schiavetti , M. Ogliastro , et al., “Co‐Detection of Respiratory Pathogens Among ILI Patients: Characterization of Samples Collected During the 2018/19 and 2019/20 Pre‐Pandemic Seasons,” BMC Infectious Diseases 24, no. 1 (2024): 881, 10.1186/s12879-024-09687-1. 39210273 PMC11361097

[39] E. L. Ciemins , A. Gillen , and M. Tallam , “RSV: A Vaccine Is Coming, Time to Educate Providers,” Vaccine 41, no. 32 (2023): 4636–4638, 10.1016/j.vaccine.2023.06.033. 37328353

[40] E. M. La , S. Bunniran , D. Garbinsky , et al., “Respiratory Syncytial Virus Knowledge, Attitudes, and Perceptions Among Adults in the United States,” Human Vaccines & Immunotherapeutics 20, no. 1 (2024): 2303796, 10.1080/21645515.2024.2303796. 38297921 PMC10841020

