# Supplementary material for: Clinical and Socioeconomic Burden of RSV Infections Among Older Adults in Primary Care: An International Prospective Cohort Study
Source: Influenza Other Respir Viruses. 2025 Oct 11;19(10):e70174. doi: 10.1111/irv.70174 (PMC12514451; doi:10.1111/irv.70174)

# **Supplementary Materials**

[**Supplementary Materials** 1](#_Toc209437897)

[**Supplementary Methods** 2](#_Toc209437898)

[Primary care structure / organization 2](#_Toc209437899)

[Sample size considerations 2](#_Toc209437900)

[Health related quality of life 2](#_Toc209437901)

[Cost analysis 3](#_Toc209437902)

[Unit costs 5](#_Toc209437903)

[Incidence of medically attended RSV infections in primary care 6](#_Toc209437904)

[**Supplementary Table 3. Patient characteristics of RSV patients by country** 8](#_Toc209437905)

[**Supplementary Table 4. Patient characteristics of RSV patients by age** 9](#_Toc209437906)

[**Supplementary Table 5. HRQoL in RSV patients overall and by country** 10](#_Toc209437907)

[**Supplementary Table 6. HRQoL in RSV patients by age** 12](#_Toc209437908)

[**Supplementary Table 7. HRQoL in RSV vs. influenza patients** 14](#_Toc209437909)

[**Supplementary Table 8. Disease characteristics of RSV patients by country** 16](#_Toc209437910)

[**Supplementary Table 9. Disease characteristics of RSV patients by age** 17](#_Toc209437911)

[**Supplementary Table 10. Healthcare resource use in RSV patients by country** 18](#_Toc209437912)

[**Supplementary Table 11. Healthcare resource use in RSV patients by age** 19](#_Toc209437913)

[**Supplementary Table 12. Healthcare resource use RSV vs influenza (NL data only)** 20](#_Toc209437914)

[**Supplementary Table 13. Work absenteeism in RSV patients overall and by country** 21](#_Toc209437915)

[**Supplementary Table 14. Work absenteeism in RSV patients by age** 22](#_Toc209437916)

[**Supplementary Table 15. Work absenteeism in RSV-positive vs. influenza (NL data only)** 23](#_Toc209437917)

[**Supplementary Table 16. Costs of RSV episodes by age** 24](#_Toc209437918)

[**Supplementary Table 17. Costs of RSV and influenza episode (NL data only)** 25](#_Toc209437919)

[**Supplementary Table 18. Incidence of RSV infections in primary care among older adults (NL only)** 26](#_Toc209437920)

[**Supplementary Figure 1. Number of RSV and influenza positive swabs** 27](#_Toc209437921)

## **Supplementary Methods**

### **Primary care structure / organization**

The structure of primary care differs between European countries. In The Netherlands, a general practitioner (GP)-led system is being used, in which the GP serves as a gatekeeper to secondary care (including emergency departments), whereas in Italy patients may visit emergency departments without referral. Italy operates a universal healthcare system known as the Servizio Sanitario Nazionale (SSN), funded primarily through taxes. This system provides free or low-cost healthcare to all residents and legal citizens, covering essential services such as hospital stays, surgeries, and emergency care. The SSN emphasizes public healthcare, but private providers also exist, often used for quicker or specialized treatment. Italy's healthcare system is decentralized, with regions responsible for managing services.

### **Sample size considerations**

Given that this study involves a descriptive analysis encompassing multiple endpoint parameters, we used ‘disease days’ and ‘medication use’ as proxy endpoint for determining sample size. In the RESCEU adult cohort, the median number of disease days was 19 (IQR: 13-27)[1]. Assuming a normal distribution after log-transformation, the standard deviation was estimated at 0.54. With 100 RSV-positive adults, the 95% CI for the geometric mean of disease days ranged from 17.1 to 21.1 days; with 200 adults, it ranged from 17.6 to 20.5 days. The proportion of patients using medication was estimated at 28%[1]. With 100 RSV-positive adults, the 95% CI for this proportion ranged from 21.2% to 40.0%, and with 200 adults, from 23.7% to 36.9%. Based on these outcomes, approximately 100 RSV cases were expected to provide sufficient precision for estimating the relevant measures.

### **Health related quality of life**

The EQ-5D-5L instrument was used to collect HRQoL data at Day 1 (initial GP visit), Day 14 and Day 30. EQ-5D-5L contains a descriptive system and a visual analogue scale (VAS) to record a respondent’s health status on the day of the survey[13]. The EQ-5D-5L descriptive system comprises the following five dimensions, each describing a different aspect of health: mobility, self-care, usual activities, pain/discomfort and anxiety/depression. Each dimension has five response levels of severity: no problems, slight problems, moderate problems, severe problems, unable to/extreme problems. The EQ VAS records the respondent’s overall current health on a vertical visual analogue scale, where the endpoints are labelled ‘The best health you can imagine’ and ‘The worst health you can imagine’. The EQ VAS provides a quantitative measure of the patient’s perception of their overall health.

EQ-5D-5L health states can be summarised by a single summary number (index value) , which reflects a respondent’s health state is according to the preferences of the general population of a country/region. Index values facilitate the calculation of quality-adjusted life years (QALYs) that are used to inform economic evaluations of healthcare interventions. An EQ-5D-5L index value was derived by applying a formula that attaches values (weights) to each of the levels in each dimension. The EQ-5D-5L states of each patient were converted into health utility values using the corresponding country-specific value set[14–16].

In absence of pre-illness EQ-5D-5L values for our study population, we compared EQ-5D-5L index values during RSV episodes (at Day 1, Day 14 and Day 30) with country-specific population norms for older adults (**Supplementary Table 1**; for NL, ≥70 years of age; IT: ≥75 years of age; mean age of study population: 76 years). We calculated the ∆EQ-5D-5L index value by subtracting EQ-5D-5L for each adult individually from age- and country-specific population norms (**Suppl. Table 2**).

**Supplementary Table 1**. Population norms for Italy[15,16] and The Netherlands[14]

| Country | EQ-5D-5L index | | | |
| --- | --- | --- | --- | --- |
|  | **Mean** | **SD** | **Median** | **Range** |
| Netherlands |  |  |  |  |
| 60-69 | 0.869 | 0.170 | n.a. | n.a. |
| 70+ | 0.852 | 0.148 | n.a. | n.a. |
| Italy |  |  |  |  |
| 65-74 | 0.91 | 0.15 | 0.95 | -0.23,1 |
| 75+ | 0.91 | 0.13 | 0.95 | 0.47, 1 |

Abbreviations: n.a., not available.

### **Cost analysis**

Perspectives chosen in this study

According to the Dutch pharmacoeconomic guideline[2], the preferred perspectives for economic evaluations are the healthcare system and societal perspective. The healthcare system perspective encompasses all medical expenses (direct costs), including out-of-pocket expenses. The societal perspective extends this by also considering productivity losses (indirect costs) in addition to all medical expenses[3].

Unit costs

*Healthcare visits costs*

Country-specific national tariffs were used to estimate unit costs for healthcare visits, including GP consultations, home visits by GPs, out-of-hours GP consultations, emergency department visits, and medical specialist consultations (**Suppl. Table 1**). Costs of diagnostic tests (eg, CRP, X-ray) were excluded as these data were not collected. Additionally, hospitalization costs (n=2) were not included.

*Medication costs*

Country-specific national tariffs were also used to estimate unit costs for medications. For all prescribed medications, we applied pharmaceutical dispensing costs as recommended by the Dutch pharmacoeconomic guidelines. In the Netherlands, short-term prescriptions (≤ 30 days) are considered first deliveries, which necessitates including pharmaceutical care delivery costs for each “new” prescription (e.g., €13.50 instead of €6.50).

Medication use was recorded at the category level (e.g., antibiotics yes/no) without specific type, dosage, or packaging size details. Therefore, we assumed the use of the most common type in each category based on national guidelines (ref NHG guidelines, Italian guidelines). For instance, the recommended first-line treatment for LRTI in adults in the Netherlands is amoxicillin 500 mg, three times daily for five days.

For over-the-counter medications, for which no strict dosing regimen or guideline exists, we assumed the cost of one unit pack. Unit cost data were obtained from the national medication price catalog (ref Medicijnkosten.nl[4] / Italian Medicine Agency[5]). When multiple prices were reported for a medication, we used the lowest indicated price, as recommended by the Kostenhandleiding[2]. If unit cost data were unavailable in the national medication price catalog, we sourced prices from the following pharmacy websites in August 2024: Etos in the Netherlands: www.etos.nl

*Productivity losses*

Productivity loss per day was estimated based on the country-specific gross average work-day salary for 2023 (Eurostat), and the total number of days off work reported by participants.

Cost analysis

All unit costs were inflated to Euro (€) year 2023, based on harmonized indices of health sector consumer prices from Eurostat[6].

Direct costs per episode were obtained by multiplying country-specific healthcare and medication use data by the country-specific unit cost per type (**Supplementary Table 2**). Indirect costs (ie, productivity losses) per episode were obtained by multiplying workdays lost with country-specific gross average work-day salary for 2018, based on Eurostat data[7]. Direct costs represent the total costs per episode from a healthcare system perspective, whereas direct and indirect costs were summed to obtain the total cost per episode from a societal perspective.

A lognormal distribution was used to address non-normally distributed parameters. Costs per episode are reported as the mean with 95% confidence intervals (CIs), calculated using bootstrapping with 10.000 samples. Additionally, the median costs are presented along with the interquartile range (IQR).

### **Unit costs**

#### **Supplementary Table 2. Unit costs**

| **Country** | **Italy** | **Netherlands** | **Reference** |
| --- | --- | --- | --- |
| Currency | EUR (€) | EUR (€) |  |
| CPI to 2023 for healthcare visits | 1.51 (2003)  1.33 (2009)  1.15 (2021) | 1.04 (2022) | Eurostat Harmonised Indices of Consumer Prices (HICP)[6] |
| CPI to 2023 for medication | 1.21 (2016)  1.0 (2024) | 1 (2024) |  |
| CPI to 2023 for work absence | 1.18 (2018) | 1.24 (2018) |  |
| Purchasing power parities (PPP) | €0.99: 1 | €1.118: 1 | Eurostat (2023)[8] |
| **Healthcare visits** |  |  |  |
| GP consultation | €17.03 | €28.74 | IT: Garratini et al (2003)[9]  NL: Kostenhandleiding 2024[2] |
| GP home visit, and out-of-hours GP consultation^1^ | €33,57 | €38.74 | IT: Garratini et al (2003)[9]  NL: Kostenhandleiding 2024[2] |
| Medical specialist visit | €26.75 | €111.63 | IT: Nomenclatore tariffario regionale delle prestazioni specialistiche ambulatoriali (2021)[10]  NL: Kostenhandleiding (2024)[2] |
| Emergency department | €166.56 | €240.00 | IT: Cremonesi et al (2009)[11]  NL: Kostenhandleiding (2024)[2] |
| **Medication (cost per unit/pack)** |  |  |  |
| Dispensing costs^2^ | €4.74 | €12.57 | IT: Garratini et al (2016)[12]  NL: Kostenhandleiding 2024[2] |
| Bronchodilators (salbutamol) | €2.54 | €2.86 | IT: Italian Medicines Agency (2024)[5],  NL: Medicijnkosten.nl (2024)[4], Kostenhandleiding (2024)[2], Etos (2024) |
| Antibiotics (amoxicillin) | €3.30 | €1.74 |  |
| Corticosteroids inhaler (fluticasone) | €15.09 | €5.53 |  |
| Corticosteroids systemic (prednisone) | €5.93 | €1.79 |  |
| Pain medication (paracetamol) | €5.96 | €1.11 |  |
| Nasal spray (xylomethazoline) | €10.00 | €3.03 |  |
| Cough syrup (bromhexide) | €12.02 | €3.43 |  |
| **Work absence** |  |  |  |
| Daily salary^3^ | € 152.22 | € 188.88 | Eurostat Annual Gross Earnings (2018)[7] |

Abbreviations: CPI: consumer price index; EUR: Euro; GP: general practitioner;

IT: Italy; NL: Netherlands;

Reported unit costs have been adjusted for inflation to 2023 prices, and normalized to the European Union average purchasing power parity (PPP=1) using comparative price levels (CPL) published by Eurostat 2023[8]. Prices were adjusted to 2023, as the 2024 CPI and CPL were not available yet at the time of this analysis (August 2024).

^1^ Since no national tariffs for out-of-hours GP consultation were available, we assumed the same costs as for GP home visits.

^2^ Pharmaceutical dispensing costs were added to unit costs for prescribed medication.

^3^ Country-specific annual gross earnings (Eurostat, 2018[7]) divided by 262 paid working days a year

### **Incidence of medically attended RSV infections in primary care**

The Julius General Practitioners’ Network (JGPN) database consists of routine care data from a dynamic cohort of approximately 370,000 individuals registered with participating general practitioners (GPs) in the city of Utrecht and surrounding areas. This database includes over ten years of data, with patient contacts electronically recorded by GPs. Diagnoses are coded according to the International Classification of Primary Care (ICPC), and prescribed medications, including dosage, are recorded using the Anatomical Therapeutic Chemical (ATC) classification system. Additionally, GP consultations are organized into episodes, which represent a series of consultations related to a single reason for encounter (a symptom or a diagnosis). Healthcare data from the JGPN are extracted anonymously on a quarterly basis.

For our study, the participating general practices in the Netherlands were located in the same geographic area as those in the JGPN, with some also being part of the JGPN network. We extracted data on all episodes of acute respiratory infection (ARI) reported in older adults (aged ≥60 years) during the study period (November to March for the 2022-2023 and 2023-2024 RSV seasons). An ARI episode was defined as any new episode coded with specific ICPC codes listed below.

To estimate the incidence of medically attended ARI, we divided the number of ARI episodes by the total follow-up time (in months) of all registered older adults in the JGPN database. We then applied the RSV positivity rate among the Dutch cohort in our study to the ARI incidence data, to estimate the annual incidence of RSV ARI. For this, we assumed no RSV circulation outside of the RSV season.

To account for multiple GP visits related to the same ARI episode, we defined a new ARI episode as one occurring after a 28-day interval without an ARI-related ICPC-1 registration (see below). We performed sensitivity analyses using broader ICPC coding criteria. This included additional codes such as those for COPD and asthma exacerbations (see below).

**ICPC codes for defining Acute Respiratory Infections (ARI):**

- **R74** - Acute upper respiratory infection
- **R83** - Other respiratory infections
- **R80** - Influenza
- **R78** - Acute bronchitis/bronchiolitis
- **R77** - Acute laryngitis/tracheitis
- **R81** - Pneumonia

**Additional ICPC Codes included in sensitivity analysis for ARI:**

- **R02** - Shortness of breath attributed to respiratory causes
- **R03** - Wheezing
- **R04** - Cough
- **R95** - Emphysema/COPD with prescription of prednisone or an antibiotic (ATC code) within 7 days before or after the episode, to ensure it represents an exacerbation rather than routine follow-up
- **R96** - Asthma with prescription of prednisone or an antibiotic (ATC code) within 7 days before or after the episode, to ensure it represents an exacerbation rather than routine follow-up

## **Supplementary Table 3. Patient characteristics of RSV patients by country**

|  | The Netherlands  N=43 | Italy  N=48 | p-value |
| --- | --- | --- | --- |
|  |  |  |  |
| Age, y, % |  |  | 0.99 |
| 60-69 | 9 (20.9%) | 10 (20.8%) | **-** |
| 70-79 | 21 (48.8%) | 23 (47.9%) | **-** |
| 80+ | 13 (30.2%) | 15 (31.3%) | **-** |
| Age, y, mean | 75.5 (8.2) | 76.9 (7.7) | 0.40 |
| Female sex | 26 (60.5%) | 27 (56.3%) | 0.68 |
| Chronic disease, any^1^ | 27/43 (62.8%) | 30/48 (62.5%) | 0.98 |
| Respiratory disease | 9 (20.9%) | 12 (25.0%) | 0.65 |
| Asthma | 3 (7.0%) | 3 (6.3%) | 1.00 |
| COPD | 8 (18.6%) | 4 (8.3%) | 0.15 |
| Cardiovascular disease | 11 (25.6%) | 12 (25.0%) | 0.95 |
| Neurological disease | 1 (2.3%) | 2 (4.2%) | 1.00 |
| Rheumatic disease | 2 (4.7%) | 2 (6.1%) | 1.00 |
| Malignancy | 1 (2.3%) | 1 (2.1%) | 1.00 |
| Diabetes mellitus | 3 (7.0%) | 8 (16.7%) | 0.16 |
| Smoking |  |  | 0.15 |
| Former | 14/41 (34.1%) | 16/48 (33.3%) | - |
| Current | 5/41 (12.2%) | 1/48 (2.1%) | - |
| Employment |  |  |  |
| Currently employed | 10/42 (23.8%) | 4/44 (9.1%) | 0.03 |
| Chronic medication use |  |  |  |
| Prednisone use | 1/43 (2.3%) | 0/15 (0.0%) | 1.00 |
| Respiratory medicine | 12/43 (27.9%) | 3/15 (20.0%) | 0.74 |
| Vaccination status |  |  |  |
| Influenza vaccine^2^ | 32/42 (76.2%) | 36/42 (85.7%) | 0.27 |
| COVID vaccine^3^ | 34/42 (81.0%) | 46/48 (95.8%) | 0.07 |
| Pneumococcal vaccine | 19/42 (45.2%) | 19/28 (67.9%) | 0.06 |

COPD: chronic obstructive pulmonary disease;

^1^ Cardio-vascular diseases included heart failure, angina, infarction, arrhythmias and structural heart diseases; neurological disease included Parkinson’s disease, epilepsy, multiple sclerosis, Alzheimer’s disease. Rheumatic disease included rheumatic arthritis, Bechterew, polymyalgia rheumatica, fibromyalgia. Malignancy included active malignant conditions, excluding non-metastatic skin cancer; diabetes mellitus was defined as type 1, type 2 or unspecified diabetes. Hypertension was not considered a chronic disease.

^2^ Influenza vaccination in the past six months

^3^ At least one COVID-vaccination

## **Supplementary Table 4. Patient characteristics of RSV patients by age**

|  | 60-74 years  N=40 | ≥75 years  N=51 | p-value |
| --- | --- | --- | --- |
| Female sex | 26 (65.0%) | 27 (52.9%) | 0.25 |
| Chronic disease, any^1^ | 19 (47.5%) | 38 (74.5%) | 0.008 |
| Respiratory disease | 5 (12.5%) | 16 (31.4%) | 0.034 |
| Asthma | 2 (5.0%) | 4 (7.8%) | 0.69 |
| COPD | 3 (7.5%) | 9 (17.6%) | 0.16 |
| Cardiovascular disease | 4 (10%) | 19 (37.3%) | 0.003 |
| Neurological disease | 1 (2.5%) | 2 (3.9%) | 1.00 |
| Rheumatic disease* | 1/76 (3.0%) | 3/43 (7.0%) | 0.63 |
| Malignancy | 1 (2.5%) | 1 (2.0%) | 1.00 |
| Diabetes mellitus | 4 (10.0%) | 7 (13.7%) | 0.75 |
| Smoking |  |  | 0.016 |
| Former | 12/39 (30.8%) | 18/50 (36.0%) |  |
| Current | 6/39 (15.4%) | 0/50 (0.0%) |  |
| Employment |  |  | 0.002 |
| Currently employed | 12/37 (32.4%) | 2/49 (4.1%) |  |
| Retired | 24/37 (64.9%) | 45/49 (91.8%) |  |
| Chronic medication use |  |  |  |
| Prednisone use | 0/27 (0.0%) | 1/31 (3.2%) | 1.00 |
| Respiratory medicine | 5/27 (18.5%) | 10/31 (32.3%) | 0.23 |
| Vaccination status |  |  |  |
| Influenza vaccine^2^ | 26/36 (72.2%) | 42/48 (87.5%) | 0.08 |
| COVID vaccine^3^ | 33/39 (84.6%) | 47/51 (92.2%) | 0.23 |
| Pneumococcal vaccine^4^ | 17/31 (54.8%) | 21/39 (53.8%) | 0.93 |

^1^ Cardio-vascular diseases included heart failure, angina, infarction, arrhythmias and structural heart diseases; neurological disease included Parkinson’s disease, epilepsy, multiple sclerosis, Alzheimer’s disease. Rheumatic disease included rheumatic arthritis, Bechterew, polymyalgia rheumatica, fibromyalgia. Malignancy included active malignant conditions, excluding non-metastatic skin cancer; diabetes mellitus was defined as type 1, type 2 or unspecified diabetes. Hypertension was not considered a chronic disease.

^2^ Influenza vaccination in the past six months

^3^ At least one COVID-vaccination

## **Supplementary Table 5. HRQoL in RSV patients overall and by country**

|  | Total  N=57 | The Netherlands  N=42 | Italy*  N=15 | *p*-value |
| --- | --- | --- | --- | --- |
| Day-1 (GP visit) |  |  |  |  |
| Any problems in |  |  |  |  |
| Mobility | 26/57 (45.6%) | 21/42 (50.0%) | 5/15 (33.3%) | 0.27 |
| Selfcare | 15/57 (26.3%) | 12/42 (28.6%) | 3/15 (20.0%) | 0.52 |
| Daily activities | 30/37 (52.6%) | 25/42 (59.5%) | 5/15 (33.3%) | 0.08 |
| Discomfort or pain | 40/57 (70.2%) | 33/42 (78.6%) | 7/15 (46.7%) | 0.02 |
| Anxiety or sadness | 9/57 (15.8%) | 7/42 (16.7%) | 2/15 (13.3%) | 0.76 |
| EQ-5D-5L utility values |  |  |  |  |
| Mean | 0.76 (0.22) | 0.72 (0.23) | 0.87 (0.13) | 0.018 |
| Median | 0.82 (0.64-0.89) | 0.82 (0.62-0.86) | 0.90 (0.77-1.00) | 0.024 |
| ∆EQ-5D-5L^1^ |  |  |  |  |
| Mean | -0.11 (0.21)** | -0.14 (0.23)** | -0.04 (0.13) | 0.12 |
| EQ-visual analogue scale |  |  |  |  |
| Mean | 33.2 (26.8) | 33.2 (26.8) | n/a | - |
| Median | 40 (5.75-60.0) | 40 (5.75-60.0) | n/a | - |
| Day-14 |  |  |  |  |
| Any problems in |  |  |  |  |
| Mobility | 18/54 (33.3%) | 14/40 (35.0%) | 4/14 (28.6%) | 0.75 |
| Selfcare | 6/54 (11.1%) | 4/40 (10.0%) | 2/14 (14.3%) | 0.64 |
| Daily activities | 26/54 (48.1%) | 21/40 (52.5%) | 5/15 (35.7%) | 0.36 |
| Discomfort or pain | 24/64 (44.4%) | 22/40 (55.0%) | 2/14 (14.3%) | 0.008 |
| Anxiety or sadness | 7/54 (13.0%) | 3/40 (7.5%) | 4/14 (28.6%) | 0.07 |
| EQ-5D-5L utility values |  |  |  |  |
| Mean | 0.85 (0.17) | 0.84 (0.18) | 0.89 (0.14) | 0.33 |
| Median | 0.89 (0.81-1.00) | 0.87 (0.81-0.92) | 0.95 (0.81-1.00) | 0.44 |
| ∆EQ-5D-5L^1^ |  |  |  |  |
| Mean | -0.02 (0.17) | -0.02 (0.18) | -0.02 (0.14) | 0.96 |
| EQ-5D-5L |  |  |  |  |
| EQ-visual analogue scale |  |  |  |  |
| Mean | 72.9 (16.2) | 68.3 (14.3) | 78.8 (16.7) | 0.006 |
| Median | 74.0 (60.0-85.0) | 70 (60.0-78.3) | 80.0 (65.0-90.0) | 0.006 |
| Day-30 |  |  |  |  |
| Any problems in |  |  |  |  |
| Mobility | 13/52 (25.0%) | 10/38 (26.3%) | 3/14 (21.4%) | 1.00 |
| Selfcare | 4/52 (7.7%) | 3/38 (7.9%) | 1/14 (7.1%) | 1.00 |
| Daily activities | 13/52 (25.0%) | 9/38 (23.7%) | 4/14 (28.6%) | 0.73 |
| Discomfort or pain | 16/52 (30.8%) | 13/38 (34.2%) | 3/14 (21.4%) | 0.51 |
| Anxiety or sadness | 7/52 (13.5%) | 4/38 (10.5%) | 3/14 (21.4%) | 0.37 |
| EQ-5D-5L utility values |  |  |  |  |
| Mean | 0.90 (0.15) | 0.90 (0.15) | 0.91 (0.15) | 0.89 |
| Median | 1.0 (0.85-1.0) | 1.00 (0.85-1.0) | 1.00 (0.78 – 1.0) | 0.78 |
| ∆EQ-5D-5L^1^ |  |  |  |  |
| Mean | 0.03 | 0.04 (0.15) | -0.00 (0.15) | 0.32 |
| EQ-visual analogue scale |  |  |  |  |
| Mean | 79.4 (14.8) | 75.0 (14.5) | 85.3 (13.4) | 0.004 |
| Median | 80 (70.0-90.0) | 77 (70.0-85.0) | 90 (80.0-99.0) | 0.003 |

^1^ ∆EQ-5D-5L reflects the difference in utility values compared to normative reference values.

*p <0.05 compared to population norm

** p<0.005 compared to population norm

## **Supplementary Table 6. HRQoL in RSV patients by age**

|  | 60-74 years | ≥75 years | *p*-value |
| --- | --- | --- | --- |
| Day-1 (GP visit) | **N=26** | **N=31** |  |
| Any problems in |  |  |  |
| Mobility | 6/26 (23.1%) | 20/31 (64.5%) | 0.002 |
| Selfcare | 4/26 (15.4%) | 11/31 (35.5%) | 0.13 |
| Daily activities | 12/26 (46.2%) | 18/31 (58.1%) | 0.37 |
| Discomfort or pain | 19/26 (73.1%) | 21/31 (67.7%) | 0.66 |
| Anxiety or sadness | 5/26 (19.2%) | 4/31 (12.9%) | 0.72 |
| EQ-5D-5L utility values |  |  |  |
| Mean | 0.77 (0.22) | 0.75 (0.22) | 0.40 |
| Median | 0.82 (0.62-0.93) | 0.81 (0.68-0.88) | 0.64 |
| ∆EQ-5D-5L^1^ |  |  |  |
| Mean (SD) | -0.10 (0.22)* | -0.11 (0.21)** | 0.86 |
| EQ-visual analogue scale |  |  |  |
| Mean | 25.8 (26.1) | 39.3 (26.4) | 0.11 |
| Day-14 | N=25 | N=29 |  |
| Any problems in |  |  |  |
| Mobility | 4/25 (16.0%) | 14/29 (48.3%) | 0.012 |
| Selfcare | 1/25 (4.0%) | 5/29 (17.2%) | 0.20 |
| Daily activities | 10/25 (40.0%) | 16/29 (55.2%) | 0.27 |
| Discomfort or pain | 12/25 (48.0%) | 12/29 (41.4%) | 0.63 |
| Anxiety or sadness | 3/25 (12.0%) | 4/29 (13.8%) | 1.00 |
| EQ-5D-5L utility values |  |  |  |
| Mean | 0.89 (0.12) | 0.82 (0.20) | 0.13 |
| Median | 0.89 (0.85-1.00) | 0.85 (0.79-0.96) | 0.18 |
| ∆EQ-5D-5L^1^ |  |  |  |
| Mean | 0.02 (0.11) | -0.05 (0.20) | 0.15 |
| EQ-visual analogue scale |  |  |  |
| Mean | 75.1 (14.9) | 71.2 (17.1) | 0.33 |
| Day-30 | N=24 | N=19 |  |
| Any problems in |  |  |  |
| Mobility | 4/24 (16.7%) | 9/28 (32.1%) | 0.20 |
| Selfcare | 1/24 (4.2%) | 3/28 (10.7%) | 0.38 |
| Daily activities | 8/24 (33.3%) | 5/28 (17.9%) | 0.22 |
| Discomfort or pain | 8/24 (33.3%) | 8/28 (28.6%) | 0.71 |
| Anxiety or sadness | 3/24 (12.5%) | 4/28 (14.3%) | 0.85 |
| EQ-5D-5L utility values |  |  |  |
| Mean | 0.92 (0.11) | 0.89 (0.17) | 0.44 |
| Median | 1.0 (0.82-1.0) | 1.0 (0.85-1.0) | 0.77 |
| ∆EQ-5D-5L^1^ |  |  |  |
| Mean | 0.05 (0.11) | 0.02 (0.18) | 0.50 |
| EQ-visual analogue scale |  |  |  |
| Mean | 80.7 (15.1) | 78.3 (14.7) | 0.54 |

^1^ ∆EQ-5D-5L reflects the difference in utility values compared to normative reference values.

*p <0.05 compared to population norm

** p<0.005 compared to population norm

## **Supplementary Table 7. HRQoL in RSV vs. influenza patients**

|  | RSV^1^ | Influenza^1^ | *p*-value |
| --- | --- | --- | --- |
| Day-1 (GP visit) | **N=57** | **N=64** |  |
| Any problems in |  |  |  |
| Mobility | 25/56 (44.6%) | 27/64 (42.2%) | 0.79 |
| Selfcare | 15/56 (26.8%) | 18/64 (28.1%) | 0.87 |
| Daily activities | 30/56 (53.6%) | 45/64 (70.3%) | 0.06 |
| Discomfort or pain | 39/56 (69.6%) | 45/64 (70.3%) | 0.94 |
| Anxiety or sadness | 8/56 (14.3%) | 13/64 (20.3%) | 0.39 |
| EQ-5D-5L utility values |  |  |  |
| Mean | 0.76 (0.22) | 0.70 (0.26) | 0.17 |
| Median | 0.82 (0.64-0.89) | 0.80 (0.58-0.89) | 0.27 |
| ∆EQ-5D-5L^2^ |  |  |  |
| Mean (SD) | -0.11 (0.21)** | -0.18 (0.25)** | 0.12 |
| EQ-visual analogue scale |  |  |  |
| Mean | 32.6 (26.9) | 42.0 (20.2) | 0.07 |
| Day-14^3^ | N=39 | N=41 |  |
| Any problems in |  |  |  |
| Mobility | 14/39 (35.9%) | 13/41 (31.7%) | 0.69 |
| Selfcare | 4/39 (10.3%) | 2/41 (4.9%) | 0.36 |
| Daily activities | 20/39 (51.3%) | 23/41 (56.1%) | 0.67 |
| Discomfort or pain | 21/39 (53.8%) | 23/41 (56.1%) | 0.84 |
| Anxiety or sadness | 3/39 (8.8%) | 10/41 (24.4%) | 0.07 |
| EQ-5D-5L utility values |  |  |  |
| Mean | 0.85 (0.17) | 0.84 (0.15) | 0.89 |
| Median | 0.89 (0.81-1.0) | 0.85 (0.78 – 0.92) | 0.36 |
| ∆EQ-5D-5L^2^ |  |  |  |
| Mean | -0.02 (0.18) | 0.03 (0.10) | 0.90 |
| EQ-visual analogue scale |  |  |  |
| Mean | 68.5 (14.4) | 71.1 (14.1) | 0.41 |
| Day-30^3^ | N=37 | N=40 |  |
| Any problems in |  |  |  |
| Mobility | 10/37 (27.0%) | 13/40 (32.5%) | 0.60 |
| Selfcare | 3/37 (8.1%) | 1/40 (2.5%) | 0.27 |
| Daily activities | 9/37 (24.3%) | 16/40 (40.0%) | 0.14 |
| Discomfort or pain | 12/37 (32.4%) | 19/40 (47.5%) | 0.18 |
| Anxiety or sadness | 4/47 (10.8%) | 10/40 (25.0%) | 0.14 |
| EQ-5D-5L utility values |  |  |  |
| Mean | 0.90 (0.15) | 0.89 (0.10) | 0.61 |
| Median | 1.0 (0.85-1.0) | 0.89 (0.81-1.0) | 0.13 |
| ∆EQ-5D-5L^2^ |  |  |  |
| Mean | 0.04 (0.15) | 0.03 (0.10) | 0.87 |
| EQ-visual analogue scale |  |  |  |
| Mean | 75.4 (14.5) | 75.1 (15.3) | 0.47 |

^1^ Patients that were both RSV and influenza positive were excluded.

^2^ ∆EQ-5D-5L reflects the difference in utility values compared to normative reference values.

^3^ For influenza patients, Day-14 and Day-30 were only collected in the Netherlands.

** p<0.001 compared to population norm

## **Supplementary Table 8. Disease characteristics of RSV patients by country**

|  | Total | The Netherlands | Italy | *p*-value |
| --- | --- | --- | --- | --- |
| Days before doctor visit |  |  |  |  |
| Mean (SD) | 5.7 (4.8) | 6.7 (5.3) | 4.9 (4.2) | 0.08 |
| Duration of illness, days |  |  |  |  |
| Mean (SD) | 16.8 (9.5) | 19.6 (8.8) | 11.8 (8.7) | <0.001 |
| Day-1 (GP visit) | N=90 | N=42 | N=48 |  |
| Dyspnea | 32 (35.6%) | 17 (40.5%) | 15 (31.3%) | 0.36 |
| Wheezing | 23/57 (40.4%) | 19/42 (45.2%) | 4/15 (26.7%) | 0.21 |
| Coughing, any | 88 (97.8%) | 42 (100.0%) | 46 (95.8%) | 0.18 |
| Productive cough | 55 (61.1%) | 28 (66.7%) | 27 (56.3%) | 0.31 |
| Sore throat | 36 (40.0%) | 17 (40.5%) | 19 (39.6%) | 0.93 |
| Coryza | 77 (85.6%) | 38 (90.5%) | 39 (81.3%) | 0.21 |
| Fever ≥ 38 ˚C | 27 (30.0%) | 13 (31.0%) | 14 (29.2%) | 0.54 |
| Muscle pain | 21/57 (36.8%) | 15 (35.7%) | 6/15 (40.0%) | 0.77 |
| Headache | 22/57 (38.6%) | 18 (42.9%) | 4/15 (26.7%) | 0.27 |
| Disturbed sleep | 28/42 (66.7%) | 27 (66.7%) | n/a | n/a |
| Fatigue | 38/71 (53.5%) | 16/23 (69.6%) | 22/48 (45.8%) | 0.06 |
| Loss of appetite | 14/71 (19.7%) | 8/23 (34.8%)%) | 6/48 (12.5%) | 0.052 |
| Day-14 |  |  |  |  |
| Not able to perform daily activities due to current symptoms^1^ | 7/67 (10.4%) | 5/22 (22.7%) | 2/45 (4.4%) | 0.02 |
| Any remaining symptoms | 51/86 (59.3%) | 37/41 (90.2%) | 14/45 (31.1%) | <0.001 |
| Day-30 |  |  |  |  |
| Not able to perform daily activities due to current symptoms^1^ | 5/64 (7.8%) | 2/21 (9.5%) | 3/43 (7.0%) | 0.01 |
| Any remaining symptoms | 37/82 (45.1%) | 24/39 (61.5%) | 13/43 (30.2%) | 0.004 |

n/a = not available

^1^ For Dutch patients, this question was only asked in the second season (2023-2024).

## **Supplementary Table 9. Disease characteristics of RSV patients by age**

|  | 60-74 years | ≥75 years | *p*-value |
| --- | --- | --- | --- |
| Days before doctor visit |  |  |  |
| Mean (SD) | 6.2 (4.3) | 5.3 (5.3) | 0.43 |
| Median (IQR) | 5.0 (3.0-9.0) | 4 (3.0-7.0) | 0.15 |
| Duration of illness, days |  |  |  |
| Mean (SD) | 17.9 (10.1) | 16.1 (9.0) | 0.48 |
| Median IQR) | 19.0 (7.0-28.0) | 15.0 (9.0-25.0) | 0.53 |
| Symptoms at day-1 (GP visit) |  |  |  |
| Dyspnea | 11/39 (28.2%) | 21/51 (41.2%) | 0.20 |
| Wheezing* | 9/26 (34.6%) | 14/31 (54.2%) | 0.42 |
| Coughing, any | 39/39 (100%) | 49/51 (96.1%) | 0.21 |
| Productive cough | 29/29 (74.4%) | 26/51 (51.0%) | 0.02 |
| Sore throat | 17/39 (43.6%) | 19/51 (37.3%) | 0.54 |
| Coryza | 35/39 (89.7%) | 42/51 (82.4%) | 0.32 |
| Fever ≥ 38 ˚C | 12/39 (30.8%) | 15/51 (29.4%) | 0.68 |
| Muscle pain | 11/26 (42.3%) | 10/31 (32.3%) | 0.43 |
| Headache | 13/26 (50.0%) | 9/31 (29.0%) | 0.49 |
| Disturbed sleep | 13/19 (68.4%) | 15/23 (65.2%) | 0.83 |
| Fatigue | 18/29 (62.1%) | 20/42 (47.6%) | 0.23 |
| Loss of appetite | 3/29 (10.3%) | 11/42 (26.2%) | 0.13 |
| Symptoms day-14 |  |  |  |
| Any remaining symptoms | 21/38 (56.8%) | 30/49 (61.2%) | 0.68 |
| Day-30 |  |  |  |
| Any remaining symptoms | 18/35 (51.4%) | 19/47 (40.4%) | 0.32 |

## **Supplementary Table 10. Healthcare resource use in RSV patients by country**

|  | The Netherlands  N=43 | Italy  N=45 | p-value |
| --- | --- | --- | --- |
| Healthcare use |  |  |  |
| ≥1 repeat GP visit^1^ | 10/42 (23.8%) | 23/45 (51.1%) | 0.009 |
| Visit to GP | 9/42 (21.4%) | 20/45 (44.4%) | 0.023 |
| Home visit by GP | 4/42 (9.5%) | 4/45 (8.9%) | 0.92 |
| Out-of-hours GP visit | 2/41 (4.9%) | 0/45 (0.0%) | 0.22 |
| Total number of GP visits^2^ |  |  |  |
| Mean (SD) | 1.6 (1.4) | 1.9 (1.2) | 0.29 |
| Median (IQR) | 1.0 (1.0-1.25) | 2.0 (1.0-2.0) | 0.025 |
| Emergency department visit | 2/43 (4.7%) | 2/45 (4.4%) | 1.00 |
| Hospitalization | 1/43 (2.3%) | 1/45 (2.2%) | 1.00 |
| Medication use |  |  |  |
| Medication use, any | 36/42 (85.7%) | 40/45 (88.9%) | 0.66 |
| Prescribed medication, any | 23/42 (54.8%) | 30/45 (66.7%) | 0.26 |
| Antibiotics | 13/42 (31.0%) | 22/45 (48.9%) | 0.09 |
| Respiratory inhalers |  |  |  |
| Beta-2-sympatics | 11/42 (26.2%) | 5/45 (11.1%) | 0.07 |
| Steroid | 5/42 (11.9%) | 9/45 (20.0%) | 0.31 |
| Systemic steroids | 4/42 (9.5%) | 14/45 (31.1%) | 0.02 |
| Over-the-counter, any | 27/42 (64.3%) | 20/45 (44.4%) | 0.06 |
| Antipyretic or pain medication | 20/41 (48.8%) | 13/45 (28.9%) | 0.06 |
| Nasal spray | 9/42 (21.4%) | 2/45 (4.4%) | 0.02 |
| Cough syrup or tablets | 13/41 (31.7%) | 11/45 (24.4%) | 0.45 |

^1^ At least one visit after the initial GP visit related to this ARI episode

^2^ All GP visits (visit to GP, home visits by GP, and out-of-hours GP visits), including the initial visit, related to this episode of ARI

## **Supplementary Table 11. Healthcare resource use in RSV patients by age**

|  | 60-74 years  N=37 | ≥75 year  N=50 | *p*-value |
| --- | --- | --- | --- |
| Healthcare use |  |  |  |
| ≥1 repeat GP visit^1^ | 12/37 (32.4%) | 21/50 (42.0%) | 0.36 |
| Number GP visits^2^ |  |  |  |
| Mean (SD) | 1.4 (0.7) | 1.9 (1.6) | 0.07 |
| Median (IQR) | 1.0 (1.0-2.0) | 1.0 (1.0-2.0) | 0.22 |
| Emergency department visit | 2/38 (5.3%) | 2/50 (4.0%) | 1.00 |
| Hospitalization | 1/38 (2.6%) | 1/50 (2.0%) | 1.00 |
| Medication use |  |  |  |
| Medication use, any | 34/37 (91.9%) | 42/50 (84.0%) | 0.27 |
| Prescribed medication, any | 23/37 (62.2%) | 30/50 (60.0%) | 0.84 |
| Antibiotics | 14/37 (37.8%) | 21/50 (42.0%) | 0.70 |
| Respiratory inhalers |  |  |  |
| Beta-2-sympatics | 7/37 (18.9%) | 9/50 (18.0%) | 0.91 |
| Steroid | 6/37 (16.2%) | 8/50 (16.0%) | 0.98 |
| Systemic steroids | 6/37 (16.2%) | 12/50 (24.0%) | 0.38 |
| Over-the-counter, any*** | 22/37 (59.5%) | 25/50 (50.0%) | 0.38 |
| Antipyretic or pain medication | 15/37 (40.5%) | 18/49 (36.7%) | 0.72 |
| Nasal spray | 5/37 (13.5%) | 6/50 (12.0%) | 1.00 |
| Cough syrup or tablets | 11/37 (29.7%) | 13/49 (26.5%) | 0.74 |

^1^ At least one visit after the initial GP visit related to this ARI episode

^2^ All GP visits (visit to GP, home visits by GP, and out-of-hours GP visits), including the initial visit, related to this episode of ARI

## **Supplementary Table 12. Healthcare resource use RSV vs influenza (NL data only)**

|  | RSV^1^ | Influenza^1^ | *p*-value |
| --- | --- | --- | --- |
| Healthcare use |  |  |  |
| ≥1 repeat GP visit^2^ | 10/41 (24.4%) | 14/43 (32.6%) | 0.41 |
| Number GP visits^3^ |  |  |  |
| Mean (SD) | 1.5 (1.4) | 1.7 (1.6) | 0.63 |
| Median (IQR) | 1.0 (1.0-1.5) | 1.0 (1.0-2.0) | 0.36 |
| Emergency department visit | 2/42 (4.8%) | 2/43 (4.7%) | 1.00 |
| Hospitalization | 1/42 (2.4%) | 1/43 (2.3%) | 1.00 |
| Medication use |  |  |  |
| Medication use, any | 35/41 (85.4%) | 38/43 (88.4%) | 0.68 |
| Prescribed medication, any | 23/41 (56.1%) | 22/43 (51.2%) | 0.65 |
| Antibiotics | 13/41 (31.7%) | 10/43 (23.3%) | 0.39 |
| Respiratory inhalers |  |  |  |
| Beta-2-sympatics | 11/41 (26.8%) | 13/43 (30.2%) | 0.73 |
| Steroid | 5/41 (12.2%) | 8/43 (18.6%) | 0.42 |
| Systemic steroids | 4/41 (9.8%) | 7/43 (16.3%) | 0.38 |
| Over-the-counter, any | 26/41 (63.4%) | 27/43 (62.8%) | 0.95 |
| Antipyretic or pain medication | 20/40 (50.0%) | 24/43 (55.8%) | 0.60 |
| Nasal spray | 9/41 (22.0%) | 20/43 (46.5%) | 0.018 |
| Cough syrup or tablets | 12/40 (30.0%) | 14/43 (32.6%) | 0.80 |

^1^ In this subgroup analysis comparing RSV to influenza, cases that were both RSV and influenza positive were excluded (n=2).

^2^ At least one visit after the initial GP visit related to this ARI episode

^3^ All GP visits (visit to GP, home visits by GP, and out-of-hours GP visits), including the initial visit, related to this episode of ARI

## **Supplementary Table** **13. Work absenteeism in RSV patients overall and by country**

|  | Total | The Netherlands | Italy | *p*-value |
| --- | --- | --- | --- | --- |
| Work status |  |  |  |  |
| Currently employed | 14/86 (16.3%) | 10/42 (23.8%) | 4/44 (9.1%) | 0.03 |
| Work absence |  |  |  |  |
| Any work absence, n (%) |  |  |  |  |
| Among those who work | 8/14 (57.1%) | 6/10 (60.0%) | 2/4 (50.0%) | 0.73 |
| Among all | 8/85 (9.4%) | 6/41 (14.6%) | 2/44 (4.5%) | 0.11 |
| Number of missed workdays |  |  |  |  |
| Among those who work |  |  |  |  |
| Mean (SD) | 4.3 (1.7) | 5.2 (5.9) | 5.8 (9.6) | 0.90 |
| Median (IQR) | 1 (0-9) | 1 (0-11) | 1.5 (0-15.8) | 0.95 |
| Among all |  |  |  |  |
| Mean (SD) | 0.87 (3.3) | 1.2 (3.6) | 0.5 (3.0) | 0.32 |
| Median (IQR) | 0 (0-0) | 0 (0-0) | 0 (0-0) | 0.13 |
| Work absence caretaker |  |  |  |  |
| Any work absence, n (%) |  |  |  |  |
| Among all | 4/67 (6.0%) | 2/41 (4.9%) | 2/26 (7.7%) | 0.64 |
| Number of missed workdays |  |  |  |  |
| Mean (SD) | 0.39 (1.9) | 0.54 (2.5) | 0.15 (0.5) | 0.43 |
| Median (IQR) | 0 (0-0) | 0 (0-0) | 0 (0-0) | 0.68 |
| Total work absence |  |  |  |  |
| Number of missed workdays |  |  |  |  |
| Mean (SD) | 1.2 (4.2) | 1.8 (5.2) | 0.6 (3.1) | 0.22 |
| Median (IQR) | 0 (0-0) | 0 (0-0) | 0 (0-0) | 0.41 |

## **Supplementary Table 14. Work absenteeism in RSV patients by age**

|  | 60-74 years | ≥75 years | *p*-value |  |
| --- | --- | --- | --- | --- |
| Work status | 12/37 (32.4%) | 2/49 (4.1%) | 0.002 |  |
| Currently employed |  |  |  |  |
| Work absenteeism |  |  |  |  |
| Any work absenteeism |  |  |  |  |
| Among those who work | 7/12 (58.3%) | 1/2 (50.0%) | 1.00 |  |
| Among all | 7/37 (18.9%) | 1/48 (2.1%) | 0.019 |  |
| Number of missed workdays |  |  |  |  |
| Among all |  |  |  |  |
| Mean (SD) | 1.6 (4.4) | 0.3 (2.0) | 0.06 |  |
| Median (IQR) | 0 (0-0) | 0 (0-0) | 0.009 |  |
| Work absenteeism caretaker |  |  |  |  |
| Any work absenteeism |  |  |  |  |
| Among all | 1/29 (3.4%) | 3/38 (7.9%) | 0.63 |  |
| Number of missed workdays |  |  |  |  |
| Mean (SD) | 0.41 (2.2) | 0.37 (1.7) | 0.92 |  |
| Median (IQR) | 0 (0-0) | 0 (0-0) | 0.48 |  |
| Total work absenteeism |  |  |  |  |
| Number of missed workdays |  |  |  |  |
| Mean (SD) | 2.0 (5.1) | 0.6 (3.4) | 0.13 |  |
| Median (IQR) | 0 (0-0) | 0 (0-0) | 0.06 |  |

## **Supplementary Table 15. Work absenteeism in RSV-positive vs. influenza (NL data only)**

|  | RSV | Influenza | *p*-value |  |
| --- | --- | --- | --- | --- |
| Work status |  |  |  |  |
| Currently employed | 10/41 (24.4%) | 16/45 (35.6%) | 0.49 |  |
| Work absenteeism |  |  |  |  |
| Any work absenteeism |  |  |  |  |
| Among those who work | 6/10 (60.0%) | 6/16 (37.5%) | 0.42 |  |
| Among all | 6/40 (15.0%) | 6/44 (13.6%) | 0.86 |  |
| Number of missed workdays |  |  |  |  |
| Among those who work |  |  |  |  |
| Mean (SD) | 5.2 (5.9) | 3.7 (7.6) | 0.61 |  |
| Median (IQR) | 3.5 (0-11) | 3.5 (0-4.0) | 0.39 |  |
| Among all |  |  |  |  |
| Mean (SD) | 1.3 (3.6) | 1.3 (4.7) | 0.50 |  |
| Median (IQR) | 0 (0-0) | 0 (0-0) | 0.87 |  |
| Work absenteeism caretaker |  |  |  |  |
| Any work absenteeism |  |  |  |  |
| Among all | 2/40 (5.0%) | 2/42 (4.8%) | 1.00 |  |
| Number of missed workdays |  |  |  |  |
| Mean (SD) | 0.6 (2.4) | 0.2 (0.8) | 0.34 |  |
| Median (IQR) | 0 (0-0) | 0 (0-0) | 0.92 |  |
| Total work absenteeism |  |  |  |  |
| Number of missed workdays |  |  |  |  |
| Mean (SD) | 1.8 (5.3) | 1.4 (5.1) | 0.74 |  |
| Median (IQR) | 0 (0-0) | 0 (0-0) | 0.94 |  |

^1^ In this subgroup analysis comparing RSV to influenza, cases that were both RSV and influenza positive were excluded (n=2)

## **Supplementary Table 16. Costs of RSV episodes by age**

|  | 60-74 years | ≥75 years |
| --- | --- | --- |
| Healthcare sector costs^1^ |  |  |
| Mean (95% CI) | €69.53  (66.00-73.20) | €85.24  (81.36-89.26) |
| Median (IQR) | €42.15  (34.66-66.18) | €50.93  (34.66-94.36) |
| Societal costs^2^ |  |  |
| Mean (95% CI) | €408.10  (361.69-459.91) | €182.69  (160.86-207.47) |
| Median (IQR) | €47.18  (34.80-102.50) | €52.37  (34.51-95.51) |

Conﬁdence intervals of means were calculated by using bootstrapping (10,000 bootstrap samples). Country-specific cost calculations were based on country-specific healthcare use and national unit prices. Unit costs were adjusted for inflation to 2023 prices, and normalized to the European Union average purchasing power parity (PPP=1) using comparative price levels (CPL) published by Eurostat 2023[8].

^1^ Healthcare sector costs do not include hospitalization costs.

^2^ Societal costs include productivity losses resulting from workdays lost by the patient as well as by a caretaker.

## **Supplementary Table 17. Costs of RSV and influenza episode (NL data only)**

|  | RSV | Influenza^1^ |
| --- | --- | --- |
| Healthcare sector costs^2^ |  |  |
| Mean (95% CI) | €77.95  (73.81-82.17) | €83.58  (79.84-87.49) |
| Median (IQR) | €44.17  (33.82-66.11) | €62.11  (35.52-76.61) |
| Societal costs^3^ |  |  |
| Mean (95% CI) | €408.24  (359.20-463.40) | €352.02  (307.89-402.32) |
| Median (IQR) | €48.13  (33.82-100.85) | €63.16  (40.13-147.56) |

Conﬁdence intervals of means were calculated by using bootstrapping (10,000 bootstrap samples). Country-specific cost calculations were based on country-specific healthcare use and national unit prices, adjusted for PPP.

^1^ In this subgroup analysis comparing RSV to influenza, cases that were both RSV and influenza positive were excluded (n=2)

^2^ Healthcare sector costs do not include hospitalization costs.

^3^ Societal costs include productivity losses resulting from workdays lost by the patient as well as by a caretaker.

## **Supplementary Table 18. Incidence of RSV infections in primary care among older adults (NL only)**

|  | Number of  ARI | Estimated number of  RSV-ARI^1^ | Follow-up time  in person-years | ARI incidence rate per 1000 person-years  (95%CI) | RSV-ARI incidence rate per 1000 person-years  (95%CI) |
| --- | --- | --- | --- | --- | --- |
| Main analysis |  |  |  |  |  |
| Season 2022-2023 | 762 | 34 | 3030 | 251.2  (233.7-269.7) | 11.2  (7.8-15.7) |
| Season 2023-2024 | 237 | 10 | 1249 | 189.7  (166.3-215.5) | 8.0  (3.8-14.7) |
| Overall | 999 | 44 | 4279 | 233.4  (219.2-248.4) | 10.3  (7.5-13.8) |
| Sensitivity analysis including COPD and asthma exacerbations^1^ | | | | | |
| Season 2022-2023 | 1116 | 50 | 3030 | 367.9  (346.6-390.2) | 16.5  (12.2-21.7) |
| Season 2023-2024 | 383 | 16 | 1249 | 306.6  (276.7-338.9) | 12.8  (7.3-20.8) |
| Overall | 1499 | 66 | 1249 | 350.3  (332.8-368.5) | 15.4  (11.9-19.6) |

95%CIs were derived using a Poisson distribution.

^1^ In this sensitivity analysis, we employed broader ICPC coding criteria to detect ARI in the Julius General Practitioners’ Network Database. This included additional codes such as those for COPD and asthma exacerbations (see **Suppl. Methods** for full list of ICPC codes).

## **Supplementary Figure 1. Number of RSV and influenza positive swabs**


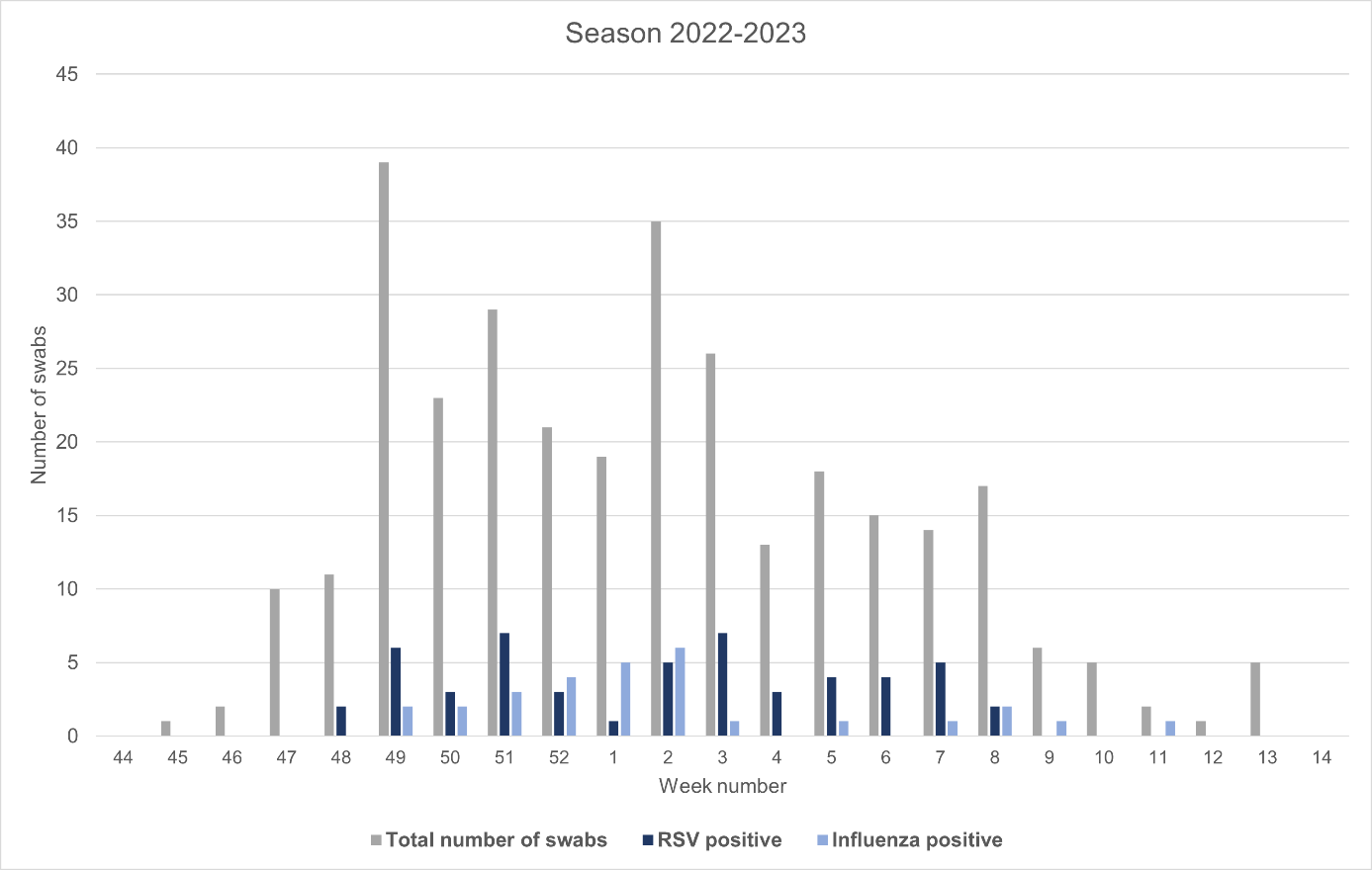

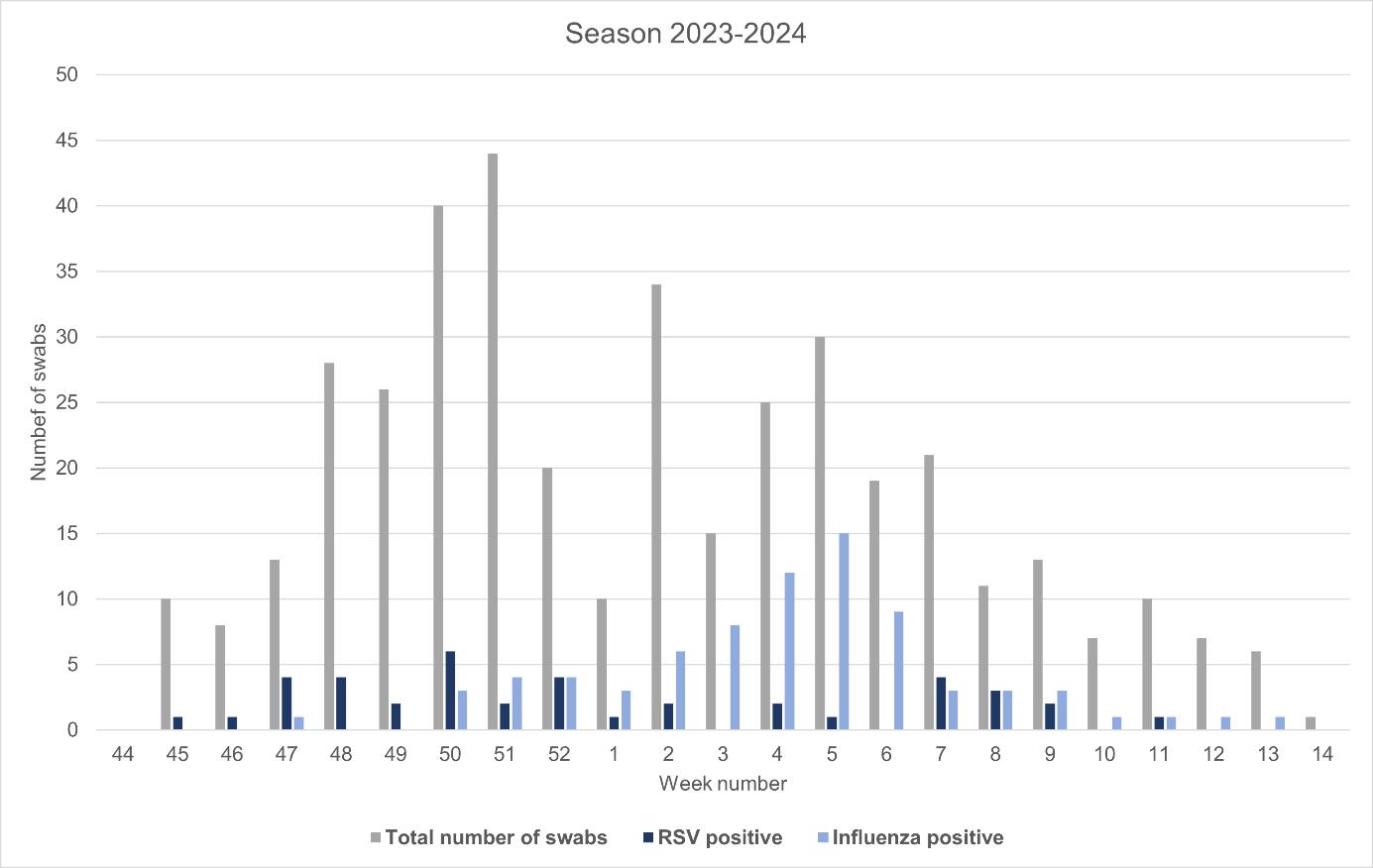

Supplement: Supplementary file 1 — Table S1: Population norms for Italy [15, 16] and The Netherlands [14]. Table S2: Unit costs. Table S3: Patient characteristics of RSV patients by country. Table S4: Patient characteristics of RSV patients by age. Table S5: HRQoL in RSV patients overall and by country. Table S6: HRQoL in RSV patients by age. Table S7: HRQoL in RSV versus influenza patients. Table S8: Disease characteristics of RSV patients by country. Table S9: Disease characteristics of RSV patients by age. Table S10: Healthcare resource use in RSV patients by country. Table S11: Healthcare resource use in RSV patients by age. Table S12: Healthcare resource use RSV versus influenza (NL data only). Table S13: Work absenteeism in RSV patients overall and by country. Table S14: Work absenteeism in RSV patients by age. Table S15: Work absenteeism in RSV‐positive versus influenza (NL data only). Table S16: Costs of RSV episodes by age. Table S17: Costs of RSV and influenza episode (NL data only). Table S18: Incidence of RSV infections in primary care among older adults (NL only). Figure S1: Number of RSV and influenza positive swabs. [file IRV-19-e70174-s001.docx]
